# Prediction of blood test values under different lifestyle scenarios using time-series electronic health record

**DOI:** 10.1371/journal.pone.0230172

**Published:** 2020-03-20

**Authors:** Takanori Hasegawa, Rui Yamaguchi, Masanori Kakuta, Kaori Sawada, Kenichi Kawatani, Koichi Murashita, Shigeyuki Nakaji, Seiya Imoto

**Affiliations:** 1 Health Intelligence Center, The Institute of Medical Science, The University of Tokyo, Minato-ku, Tokyo, Japan; 2 Human Genome Center, The Institute of Medical Science, The University of Tokyo, Minato-ku, Tokyo, Japan; 3 Department of Social Medicine, Graduate School of Medicine, Hirosaki University, Hirosaki, Aomori, Japan; 4 COI Research Initiatives Organization, Hirosaki University, Hirosaki, Aomori, Japan; Tabriz University of Medical Sciences, ISLAMIC REPUBLIC OF IRAN

## Abstract

Owing to increasing medical expenses, researchers have attempted to detect clinical signs and preventive measures of diseases using electronic health record (EHR). In particular, time-series EHRs collected by periodic medical check-up enable us to clarify the relevance among check-up results and individual environmental factors such as lifestyle. However, usually such time-series data have many missing observations and some results are strongly correlated to each other. These problems make the analysis difficult and there exists strong demand to detect clinical findings beyond them. We focus on blood test values in medical check-up results and apply a time-series analysis methodology using a state space model. It can infer the internal medical states emerged in blood test values and handle missing observations. The estimated models enable us to predict one’s blood test values under specified condition and predict the effect of intervention, such as changes of body composition and lifestyle. We use time-series data of EHRs periodically collected in the Hirosaki cohort study in Japan and elucidate the effect of 17 environmental factors to 38 blood test values in elderly people. Using the estimated model, we then simulate and compare time-transitions of participant’s blood test values under several lifestyle scenarios. It visualizes the impact of lifestyle changes for the prevention of diseases. Finally, we exemplify that prediction errors under participant’s actual lifestyle can be partially explained by genetic variations, and some of their effects have not been investigated by traditional association studies.

## Introduction

Recently, the continuously increasing cost of medical care has received significant attention. The center of the idea aimed at curbing this trend is using electronic health records (EHRs) to detect signs and preventive measures of diseases such as diabetes. EHRs consist of patient’s medical information, *e.g*., demographics, symptoms, and blood test values. Thus, while the essential purpose of EHR is to document the history of patient care for reimbursement, the accumulated EHRs have been gathering a great deal of attention for advanced purposes. For example, the analysis of EHRs enables us to facilitate clinical decision support, to predict condition-specific clinical processes, and to improve clinical treatment plans beyond traditional clinical encounters. In particular, periodic time-series EHRs collected in, *e.g*., annual medical check-ups, can clarify trends of changes in check-up results depending on lifestyle and social status. However, such time-series data have many missing observations and some results are strongly correlated to each other. These problems make the analysis difficult.

Traditionally, time-aware models, *e.g*., survival modeling, are utilized when we focus on the time-to-event to evaluate the effect of treatments or occurrence of events for some endpoints, *e.g*., the mortality of cardiovascular diseases. On the other hand, there exist statistical techniques, which utilize statistical models to mathematically formalize the generative process of the data under assumptions. They can elucidate hidden mechanism of the target system and also incorporate the effect of time such as dynamic Bayesian networks (DBNs) [1, 2] and the state space models (SSM) [3–5]. For example, Nachimuthu *et al*. [6] used DBNs to model temporal relationships between insulin and glucose homeostasis and predicted the future glucose levels of a patient admitted in an ICU. Sandri *et al*. [7] and Gatti *et al*. [8] also used DBNs with the restrictions on the causal structure to model organ failure. Similarly, Peelen *et al*. [9] used a hierarchical Bayesian strategy for modeling organ failure. These researches constructed statistical models for the analysis of target medical incident and successfully obtained clinical findings. Thus, we design statistical models that can represent relevance among check-up results, lifestyle changes, and social status depending on time.

In this paper, we focus on blood test values in EHRs and inference of the effect to blood test values by the changes of body composition values such as Body Mass Index (BMI), lifestyle, and social status. For this purpose, we apply a statistical approach using the SSM, which has successful applications in a wide range of fields [10–14]. Our proposed model is designed to infer hidden state variables, which summarize the internal (unobserved) medical states emerged in blood test values, at each time-point for each person. The model realizes the estimation of the effect of body composition values, lifestyles, and social status to blood test values. For the extraction of meaningful relationships, we suppress the sparse constraint to regulatory effect matrices and infer their parameter values by maximization of the L1 regularized likelihood. To this end, we developed a new algorithm to obtain active sets of parameters and estimate a maximizer of the L1 regularized likelihood using the EM algorithm. The proposed approach is applied to EHR obtained in the Hirosaki cohort study in Japan, which is a free participation medical check-up cohort including men and women, aged from 12 to 91 obtained from 2005 to 2015.

## Materials and methods

### Periodically collected time-series EHR

Here, we consider that EHR is a set of clinical records with date, and it includes each diagnosis or check-up result. Thus, clinical records obtained at different dates, of which individuals are the same, can be integrated to refer to participant information. Especially, in this study, we consider that EHRs consist of collected certain items at regular interval through cohort studies and regular medical check-ups. Although a predetermined interval of visits may exist for each person’s condition, *e.g*., annual medical check-ups are held at one-year intervals and pre-diabetes patients are advised to check their blood test values every few months, many participants do not actually follow these recommendations. Thus, the most striking characteristic of EHR is the irregularity of participants visits.

Let ***y***_*k*,*t*_ be *q*-dimensional observed blood test values of the *k*th (*k* = 1, …, *K*) participant at the *t*th (*t* = 1,…,*T*_*k*_) time point (we here consider the data of the first time of visit as ***y***_*k*,1_), *K* is the number of participants, and *T*_*k*_ is the final time of visit of the *k*th participant. We have a set of observation data *Y* = {*Y*_1_,…,*Y*_*K*_}, where *Y*_*k*_ = {***y***_*k*,1_,…, $y_{k,T_{k}}$} and the number of observations of the *k*th participant is not necessarily *T*_*k*_ because the *k*th participant might not visit at the *t*′th (1< *t*′ <*T*_*k*_) time-point.

### Linear state space model for EHR

The state space model (SSM) has been widely used in time-series analysis and it consists of a state transition equation and an observation equation. The state transition equation models the process of the hidden state variables and the observation equation links the observations to these underlying states. The advantage of SSM is the scalability of the design of the model. In this research, we have three motivations; (a) inference of the effect of environmental factors such as lifestyles to blood test values, (b) prediction of one’s blood test values under certain environmental factors, and (c) estimation of genetic effects as the explanatory factors for prediction errors of blood test values. Moreover, there are two major problems; (i) there are strongly correlated blood test values and (ii) EHR has several missing observations. To overcome these problems, we apply the state space model.

Let ***x***_*k*,*t*_ and ***z***_*k*,*t*_ be a series of *p*-dimensional vectors containing hidden variables representing the internal medical states emerged in blood test values and *m*-dimensional vector containing the values representing environmental factors such as body composition values of the *k*th (*k* = 1,…,*K*) participant at the *t*th (*t* = 1,…,*T*_*k*_) time point, respectively. Then, we consider a state space model for EHR represented by

$$x_{k,t}=Ax_{k,t-1}+Gz_{k,t-1}+v_{k,t},$$ (1)

$$y_{k,t}=Hx_{k,t}+w_{k,t},$$ (2)

where *A* = (***a***_1_, …, ***a***_*p*_)′ is a *p* × *p* regulatory matrix, ***a***_*i*_ = (*a*_*i*,1_, …, *a*_*i*,*p*_)′ (*i* = 1, …, *p*) is a *p*-dimensional vector including regulatory effects on the *i*th hidden variable by other ones, *G* = (***g***_1_, …, ***g***_*p*_)′ is a *p* × *m* regulatory matrix, ***g***_*i*_ = (*g*_*i*,1_, …, *g*_*i*,*m*_)′ is an *m*-dimensional vector representing their regulatory effects on the *i*th hidden variable by ***z***_*k*,*t*−1_, *H* = (***h***_1_, …, ***h***_*q*_)′ is a *q* × *p* map matrix, ***h***_*i*′_ = (*h*_*i*′,1_, …, *h*_*i*′, *p*_)′ (*i*′ = 1, …, *q*) is a *p*-dimensional vector that maps hidden variables on the *i*th element of ***y***_*k*,*t*_, ***v***_*k*,*t*_ ∈ *R*^*q*^ is a vector of system noise, and ***w***_*k*,*t*_ ∈*R*^*q*^ is a vector of observational noise. Here, diagonal elements of *A* are restricted to less than 0.8 and we set system noise as ***v***_*k*,*t*_ ∼ *N*_*p*_(**0**_*p*_, *Q*) and observation noise as ***w***_*k*,*t*_ ∼ *N*_*q*_(**0**_*q*_, *R*), where *Q* and *R* are *p* × *p* and *q* × *q* diagonal matrices. The initial state vector ***x***_*k*,0_ is assumed to be a Gaussian random vector with mean vector ***μ***_*k*,0_ and covariance matrix *Σ*_0_, *i.e*., ***x***_*k*,0_ ∼ *N*_*p*_(***μ***_*k*,0_, *Σ*_0_). Because the relationship between observation variables ***y***_*k*,*t*_ can be represented by *A* and *H* [15], we can estimate the effect from the lifestyle ***z***_*k*,*t*_ to the observation variables ***y***_*k*,*t*_ with considering the effects between observation variables ***y***_*k*,*t*_ using the proposed state space model. Note that we consider that *A*, *G* and *H* should be sparse matrices.

The SSM consisting of Eqs (1) and (2) represents a process how observation values ***y***_*k*,*t*_ are generated from an unobserved dynamic system through a latent state vector ***x***_*k*,*t*_. Our aim is to estimate posterior distributions of state vectors ***x***_*k*,*t*_ (*t* = 1,…, *T*_*k*_) given a set of observation data *Y*_*k*_ = {***y***_*k*,1_, …, $y_{k,T_{k}}$} in Bayesian-statistics manner. The estimation is carried out sequentially from *t* = 1 to *t* = *T*_*k*_ by updating the posterior distribution of ***x***_*k*,*t*_ according to the dynamics of Eq (1) followed by addition of information of ***y***_*k*,*t*_ to the set of observation data according to Eq (2) one after another. The modeling and estimation approach can naturally handle missing observations in time-series data without external imputations by the following mechanism; if the observation at time *t*′, ***y***_*k*,*t*′_ (1 < *t*′ < *T*), is a missing case, the update of posterior distribution of ***x***_*k*,*t*′_ is done according to Eq (1) only and the step to add information of ***y***_*k*,*t*′_ is just skipped; then the estimation process from *t* = *t*′+ 1 is continued without inconsistency.

### Maximum likelihood estimation using the EM algorithm with L1 regularization

We applied *L*1 regularization to select effective sets of elements for *A*, *G*, and *H*. Let {*Y*, *X*} = {*Y*_1_, …, *Y*_*K*_, *X*_1_, …, *X*_*K*_} be the complete data set, where *X*_*k*_ = {***x***_*k*,0_, …, $x_{k,T_{k}}$} is the set of state variables. Here, ***y***_*k*,*t*_ can be unobserved when the *k*th individual did not participate the check-up at time *t*, but all ***x***_*k*,*t*_ are estimated. Furthermore, let the probability densities *P*(***x***_*k*,0_) and *P*(***x***_*k*,*t*_|*x*_*k*,*t*−1_) be the *p*-dimensional Gaussian distributions *N*_*p*_(***μ***_*k*,0_, Σ_0_) and *N*_*p*_(*F****x***_*k*,*t*−1_ + *G****z***_*k*,*t*−1_, *Q*), respectively, and *P*(***y***_*k*,*t*_|***x***_*k*,*t*_) be the *q*-dimensional Gaussian distribution *N*_*q*_(***x***_*k*,*t*_, *R*). Then joint likelihood for the complete data set is given by

$$P\left(Y,X;\theta \right)=\prod_{k=1}^{K}P\left(x_{k,0}\right)\prod_{t\in T_{k}}P\left(x_{k,t}|x_{k,t-1}\right)\prod_{t\in T_{k,obs}}P\left(y_{k,t}|x_{k,t}\right),$$ (3)

where ***θ*** = {*A*, *G*, *Q*, *H*, *R*, ***μ***_1,0_, …, ***μ***_*k*,0_}, and $T_{k}$ and $T_{k,obs}$ ($T_{k,obs}\in T_{k}$) are sets of all time points and the observed time points of the *k*th participant, respectively. In this study, we used the Expectation-Maximization (EM) algorithm [16] to search for the parameter vector ***θ*** that maximizes *P*(*Y*; ***θ***) under *L*1 regularization. The *L*1 regularized log-likelihood is given by

$$\begin{array}{c} \text{log}\int \prod_{k=1}^{K}P\left(x_{k,0}\right)\prod_{t\in T_{k}}P\left(x_{k,t}|x_{k,t-1}\right)\prod_{t\in T_{k,obs}}P\left(y_{k,t}|x_{k,t}\right)dx_{1,0}\ldots dx_{K,T_{K}}\\ \;-\sum_{i=1}^{p}\sum_{j=1}^{p}\lambda_{i}^{\left(s\right)}|A_{i,j}|-\sum_{i=1}^{p}\sum_{j=1}^{m}\lambda_{i}^{\left(s\right)}|G_{i,j}|-\sum_{i^{\prime }=1}^{q}\sum_{j=1}^{p}\lambda_{i}^{\left(o\right)}\left|H_{i,j}\right|, \end{array}$$ (4)

where $\lambda_{i}^{\left(s\right)}$ and $\lambda_{i}^{\left(o\right)}$ are the *L*1 regularization terms for the *i*th row of system and observation models, respectively.

In the EM algorithm, the conditional expectation of the joint log-likelihood of the complete data set

$$q\left(\theta |\theta_{l}\right)=E\left[\text{log}\;P\left(Y,X|\theta \right)|Y;\theta_{i}\right],$$ (5)

is iteratively maximized with respect to ***θ*** until convergence, where ***θ***_*l*_ is the parameter vector obtained at the *l*th (previous) iteration. More details of the proposed algorithm are described in the supplemental materials.

Because the EM-algorithm tries to search for the parameter values that can achieve the local minimum likelihood, we set several initial values and the dimension of the hidden variables *p* to obtain better models. To select the most plausible model among the estimated results, we apply Bayesian information criterion [17] described as

BIC=-2logL(Y|θ)+df(θ)logν, (6)

$$L(Y|\theta )=\int \prod_{k=1}^{K}P\left(x_{k,0}\right)\prod_{t\in T_{k}}P\left(x_{k,t}|x_{k,t-1}\right)\prod_{t\in T_{k,obs}}P\left(y_{k,t}|x_{k,t}\right)dx_{1,0}\ldots dx_{K,T_{K}},$$ (7)

where df(***θ***) is the degree of freedom, *i.e*., the number of non-zero parameters values, and *ν* is the number of observed points. The source code is available at https://github.com/hase62.

## Results

### EHR in Hirosaki cohort study

We apply the method to the data obtained from the Hirosaki Center-of-Innovation (COI) health promotion project, which is a cohort study in Hirosaki City (population is about 10, 000) in northern Japan. Every year, about 1,000 people have been checking their health status with free participation. Since the final goal is to extend the life span, it has measured comprehensive items (total about 2, 000), such as invasive/non-invasive clinical test values, body composition values, lifestyles, medication, exercise capacity, and cognitive ability. The data actually include many missing observations because individuals did not necessarily participate all check-ups. Thus, for example, if the *k*th participant visited 2009, 2010, 2013, and 2014’s check-ups, we have *Y*_*k*_ = {***y***_*k*,1_, ***y***_*k*,2_, ***y***_*k*,5_, ***y***_*k*,6_}, where ***y***_*k*,1_ is the observation data at the first year (2009) of visit. Note that each ***y***_*k*,*t*_ (*t* = 1, 2, 5, 6) includes all features. All participants gave written informed consent and the study was approved by IRB in Hirosaki University (Num. 2017-026).

We used the data obtained from 2007 to 2015. Especially in this study, we firstly focused on 1, 196 elderly persons aged from 55 to 75 because young participants may have normal blood test values regardless of their lifestyle and we are interested in the association of the effect of lifestyle changes and blood test values in elderly people. We considered exclusion criteria that removed 533 participants who used any medicine because we are interested in the effect of lifestyle changes to the blood test values, but not the effect of medicine. We thought that the blood test values were potentially affected by the medicine. We further excluded 104 participants who participated only one medical check-up between 2007 and 2015 because we cannot obtain any time-series blood test values from them. Finally, the numbers of participants for male and female were 156 and 273, respectively, and thus 429 (35.9%) participants were remained from 1, 196 elderly participants.

For baseline information comparison, we prepared histograms of ages and target blood test values of (i) included participants, (ii) excluded participants who used any medicine, and (iii) excluded participants who participated only one medical check-up in the supplementary materials. We then evaluated the differences between (i) and (ii) datasets and between (i) and (iii) datasets by the Wilcoxon rank-sum test. In the former comparison, we hypothesized that some differences should be observed, because dataset (ii) were collected sick participants and they were not our analysis target. From the Wilcoxon rank-sum test between (i) and (ii), we confirmed the difference is significant as considered. Therefore, the participants in (ii) could be affected by using medicine for the treatment and this exclusion criterion seems to be reasonable. On the other hand, we confirmed that the comparison between (i) and (iii) indicated no significant difference (the adjusted threshold of the *p*-value is 0.05/(39 × 2) = 6.41E-4 by the Bonferroni method). Thus, no serious sample bias has been detected.

We focus on 38 blood test values in the collected check-up results and they are handled as ***y***_*k*,*t*_ and listed in Table 1. These check-up items are generally collected for the screening of chronic diseases such as Diabetes and high blood pressure. As environmental factors, we prepare body composition values [Bc], lifestyles [Lf] and social status [Ss], and they are handled as ***z***_*k*,*t*_ and listed in Table 2. Here, we used Body weight, Abdominal Circumference, W/H-ratio, BMI, and BFP as representative of body composition because they are usually used as easy indicators of obesity, exercise, diet and so on.

**Table 1:** The list of observed blood test values in *y*_*k*,*t*_.

Num.	Name	Num.	Name
y1	Systolic Blood Pressure	y20	ALT (GPT)
y2	Diastolic Blood Pressure	y21	Total Protein
y3	PWV Left-Right Average	y22	ALB (Improved BCP)
y4	ABI Left-Right Average	y23	Creatinine
y5	Bone Density (Acoustic Value)	y24	Urea Nitrogen
y6	Bone Density (Z score)	y25	Uric Acid
y7	Bone Density (T score)	y26	Total Cholesterol
y8	Serum Glucose	y27	Triglyceride (TG)
y9	HbA1c (NGSP)	y28	HDL Cholesterol
y10	Muscle Amount (Height Corrected)	y29	LDL Cholesterol
y11	White Blood Cell Count (WBC)	y30	Sodium
y12	Red Blood Cell Count (RBC)	y31	Potassium
y13	Hemoglobin	y32	Chlorine
y14	Hematocrit	y33	Calcium
y15	MCV	y34	Inorganic Phosphorus
y16	MCH	y35	Serum Iron
y17	MCHC	y36	C3
y18	Total Bilirubin	y37	C4
y19	AST (GOT)	y38	nonHDL Cholesterol

**Table 2:** List of environmental factors representing body composition values [Bc], lifestyles [Lf], and social status [Ss] handled as *z*_*k*,*t*_.

Num.	Name
z1	[Bc] Body Weight
z2	[Bc] Abdominal Circumference
z3	[Bc] Waist / Hip Ratio
z4	[Bc] Body Mass Index
z5	[Bc] Body Fat Percentage
z6	[Lf] Working Days (Days / Week)
z7	[Lf] Sleep Disorder (0:None, 1:Yes)
z8	[Lf] Current Smoking Habits (0:None, 1:Yes)
z9	[Lf] Current Drinking Habits (0:None, 1:Yes)
z10	[Lf] Sleeping Hours
z11	[Lf] Midday Nap (0:None, 1:Yes)
z12	[Lf] Averaged Exercise Hours (1:None, 2:1 Time / Week, 3:2-3 Times / Week, 4:4-5 Times / Week, 5:Everyday)
z13	[Ss] Age at Survey Date
z14	[Ss] Family Structure (#Family members)
z15	[Ss] Marital Status (0:None, 1:Yes)
z16	[Ss] Farmer (0:None, 1:Yes)
z17	[Ss] Final Educational Background (0:Elementary or Junior High or High School, 1:Junior College or Vocational School, 2:University or College)

**Fig 1:**
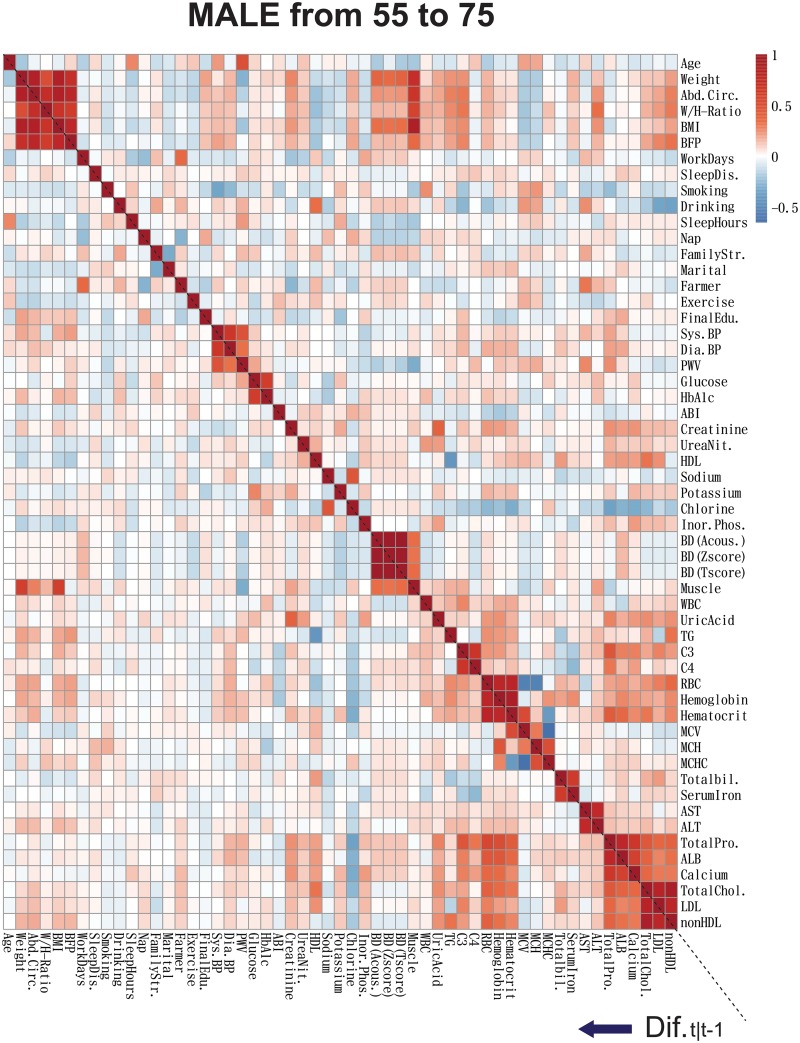
The Spearman correlation coefficients of observed blood test values and environmental factors. The bottom-left part and the upper right part of the heatmap represent the Spearman correlation coefficients among (***y***_*k*,*t*_ ‒ ***y***_*k*,*t*−1_) and (***z***_*k*,*t*_ ‒ ***z***_*k*,*t*−1_), and among ***y***_*k*,*t*_ and ***z***_*k*,*t*_ for Male time-series data, respectively.

In order to show the overall picture of the correlation among blood test values and lifestyles, the correlations of ***y***_*k*,*t*_ and ***z***_*k*,*t*_ for Male dataset are illustrated in Fig 1. Here, in order to illustrate the immediate effect of changes of environmental factors to the changes of blood test values, we illustrated the results of the spearman correlation analysis among (***y***_*k*,*t*_ ‒ ***y***_*k*,*t*−1_) and (***z***_*k*,*t*_ ‒ ***z***_*k*,*t*−1_), and ***y***_*k*,*t*_ and ***z***_*k*,*t*_. In the blood test values ***y***_*k*,*t*_, we can see that some values are highly correlated, for example, ‘HbA1c and Glucose’, and ‘Total Cholesterols, LDL, and nonHDL’. They should be represented by the same hidden variables in the proposed SSM. Similarly, body composition values are mutually highly correlated, and they are also correlated with Bone Densities (BDs), Muscle Amount, Blood Counts, Cholesterols, and so on. On the other hand, among (***y***_*k*,*t*_ ‒ ***y***_*k*,*t*−1_) and (***z***_*k*,*t*_ ‒ ***z***_*k*,*t*−1_), they have weaker correlations especially in Waist/Hip ratio (W/H-ratio) than those in ***y***_*k*,*t*_ and ***z***_*k*,*t*_. Thus, the changes of body composition values and lifestyles cannot immediately affect to blood test values even if some values suddenly change. These correlations enable us to impute missing observation data. For example, high correlations between some observation variables at time *t* and *t*−1 enable us to predict these variables at time *t*′, which is a time-point with missing observation data, from the variables at time *t*′ − 1. The details of the calculation are written in the supplemental materials.

### Inference of internal medical states and their relationship

For the above explained datasets, we applied the proposed SSM to classify the relationships among blood test values and environmental factors. We tested 50 calculations for each system dimension *p* from 3 to 15 with randomly prepared initial parameter values, and then searched for the value of *p* that achieves the lowest BIC score as described in Eq (6). Consequently, for both datasets, *p* = 14 was selected as the best dimension. The BIC score is illustrated for each of system dimensions, *p*, in the supplementary materials.

**Fig 2:**
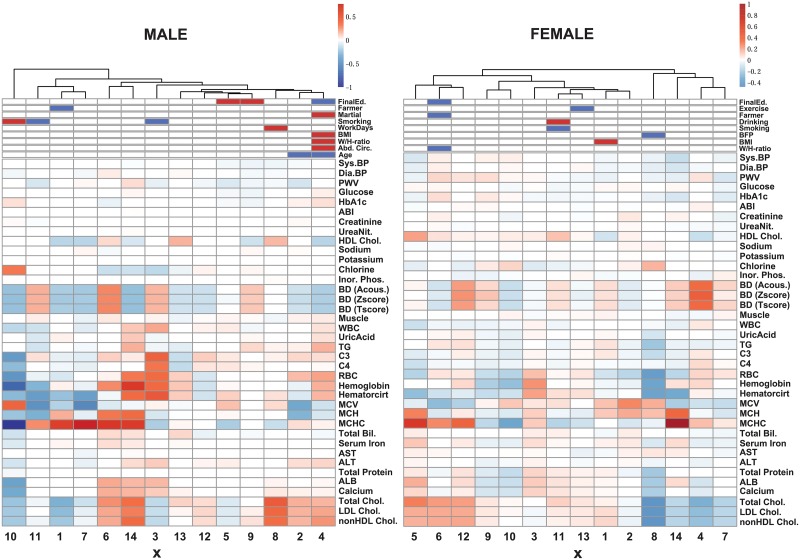
The heatmap of the effect of *z*_*k*,*t*_ to *y*_*k*,*t*_. The heatmap represents the effect of ***z***_*k*,*t*_ to ***x***_*k*,*t*_ as an estimated matrix *G* in the upper part and that of ***x***_*k*,*t*_ to ***y***_*k*,*t*_ as an estimated matrix *H* in the bottom part, and thus introduces the indirect effect from ***z***_*k*,*t*_ to ***y***_*k*,*t*_. The numbers on horizontal axis mean the element numbers of the 14-dimensional vector ***x***_*k*,*t*_. For example, the column with ‘3’ in the heatmap displays the effect from the 3rd element of the hidden variable ***x***_*k*,*t*_ to the observation variables ***y***_*k*,*t*_. For *H*, each value is normalized as *H*_*i*′, *j*_ × $\sqrt{(}\sum_{k=1}^{K}\sum_{t=1}^{T_{k}}\left(x_{k,t,j}^{2}\right))$ and then *H*_*i*′,*j*_/max(|*H*|), where *H*_*i*′,*j*_ is the *i*′th row and the *j*th column element of *H* and max(|*H*|) is the max absolute value of *H*. For *G*, positive and negative elements are illustrated as red and blue, respectively.

For each dataset, we next evaluated the effects of ***x***_*k*,*t*_ and ***z***_*k*,*t*_ to the blood test values ***y***_*k*,*t*_. The heatmaps of the estimated sparse matrices *H* and *G* are illustrated in Fig 2. This figure shows the effect of ***z***_*k*,*t*_ to ***x***_*k*,*t*_ as an estimated matrix *G* in the upper part and that of ***x***_*k*,*t*_ to ***y***_*k*,*t*_ as an estimated matrix *H*. Through the results, some clusters exist in the blood tests. We summarize here: ‘Diastolic and Systolic Blood Pressures (BPs), and PWV’ (BP Group), ‘HbA1c and Glucose’ (Diabetes Group), ‘RBC, Hemoglobin, and Hematocrit’ (RBC Group1), ‘MCH and MCV’ (RBC Group2), ‘Total Cholesterols, LDL Chol., HDL Chol, and nonHDL Chol.’ (Dyslipidemia Group), ‘Total Bilirubin and Serum Iron’ (Liver Group1), ‘ALT and AST’ (Liver Group2), and ‘C3, Total Pro., ALB, and Calcium’ (Liver Group 3). These groups are similar to the result of correlation analysis in Fig 1 and can be similarly changed in response to the changes of ***x***_*k*,*t*_ and ***z***_*k*,*t*_. Also, some hidden variables have similar effect to ***y***_*k*,*t*_ in *H* with partially different components. On the other hand, some blood test values, *e.g*., ABI and Potassium, are not or weakly regulated by ***x***_*k*,*t*_; these blood test values are robust from other blood test values, and the changes of our prepared body composition values and lifestyles. Here, we summarize the characteristics of the results in Table 3.

**Table 3:** Effect of lifestyles for blood test values. The columns (+) and (-) indicate the list of blood test values that are increased and decreased by the corresponding environmental factors in male or female, respectively.

Environmental factors	Male (+)	Male (-)	Female (+)	Female (-)
Farmer	MCV, Hematocrit, Dyslipidemia Group, and BDs	MCH, and MCHC	BDs, MCV, and Hematocrit	Dyslipidemia Group, and MCHC
Increasing Age	BDs, HDL and RBC Group2	Dyslipidemia Group, RBC Group1, and MCHC		
Smoking	Chlorine, and MCV	C3, C4, RBC, and Liver Group 3	MCHC	HDL
Drinking			HDL	MCHC
Higher Education	BDs, and RBC Group2	Dyslipidemia Group, RBC Group1, and MCHC	BDs, MCV, and Hematocrit	Dyslipidemia Group, and MCHC
Married Status	Dyslipidemia Group, and RBC Group1	BDs, HDL, and RBC Group2		
Longer Working Hours	Dyslipidemia Group			
Increasing BMI	Dyslipidemia Group, and RBC Group1	BDs, HDL and RBC Group2	Dyslipidemia Group, and RBC Group2	HDL, and RBC
Increasing W/H-ratio	Dyslipidemia Group, and RBC Group1	BDs, HDL and RBC Group2	BDs, MCV, and Hematocrit	Dyslipidemia Group, and MCHC
Increasing AC	Dyslipidemia Group, and RBC Group1	BDs, HDL and RBC Group2		
Increasing BFP			RBC Group1, TG, Dyslipidemia Group, and Liver Group3	RBC Group2, and Chlorine
Exercise				Dyslipidemia Group

For each dataset, we then evaluate the effect among ***x***_*k*,*t*_ based on the matrix *F*. Hidden variables that share similar effects in *H* seems to have feedback structures, but we cannot capture any clear structure. The regulatory relationships are illustrated in the supplemental materials.

### Scenario-based blood test value simulation

**Fig 3:**
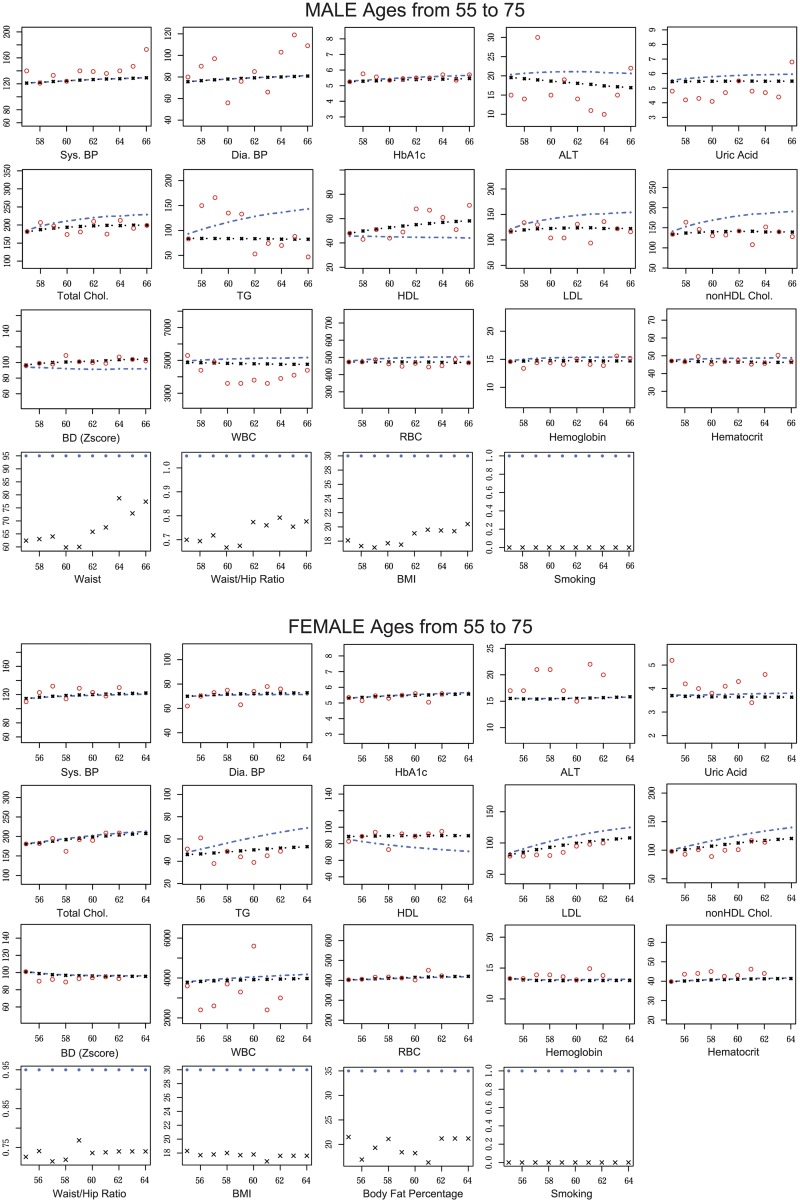
The comparison among predicted and actual blood test values of representative healthy male and female. The predicted values are calculated both under actual and unhealthy conditions for representative healthy male and female. The predicted blood test values are calculated by our estimated state space model and ***z***_*k*,*t*_ (actual and unhealthy) without filtering steps. Red circles are observed blood test values, black lines are predicted blood test values under actual condition, and blue lines are predicted blood test values under the unhealthy condition. Black crossed marks are actual ***z***_*k*,*t*_, and blue circles are postulated ***z***_*k*,*t*_ under the unhealthy condition. The horizontal and vertical axes indicate the ages of the selected participant at the medical check-up years and the blood test values, respectively. Thus, the youngest age in the horizontal axis means the participant’s age at the first year of visit to the medical check-up event. Note that, in the calculation, we consider the observation at the first year of visit as ***y***_*k*,1_.

We compare the predicted and actual time-series blood test values of participants when improving or corrupting their body composition values and lifestyles. In this experiment, we postulate two situations; (i) a healthy person suddenly corrupted their body composition values and lifestyles, and (ii) an unhealthy person started to improve their body composition values and lifestyles. At first, we extracted a healthy male and female, and predict blood test values using the estimated parameter values and (i-a) actual ***z***_*k*,*t*_ and (i-b) postulated ***z***_*k*,*t*_ to conduct them to be unhealthy persons. Here, we consider unhealthy status as Abdominal Circumference (AC) = 95 [cm], W/H-ratio = 1.05, BMI = 30, and Smoking status for male, and W/H-ratio = 0.95, BMI = 30, Body Fat Percentage (BFP) = 35, and Smoking status for female. The simulation results are illustrated in Fig 3.

In these figures, we assume that the body composition values and lifestyles suddenly changed to unhealthy condition at the first medical check-up year. From the simulations, participants in Dyslipidemia Group seem to be the most affected. The male under unhealthy condition gradually increases TG and LDL and finally becomes Dyslipidemia when he is 64 years old. In contrast to the postulated unhealthy condition, actual status at 64 years old is not Dyslipidemic. Furthermore, ALT and Uric Acid are weakly increasing to suspected areas, and HbA1c, BDs, WBC, RBC, Hemoglobin, and Hematocrit are also weakly increasing in the Male in contrast that they are almost not varied in the Female. In both persons, BPs and HbA1c are not affected by the changes in body composition values and lifestyles used here.

**Fig 4:**
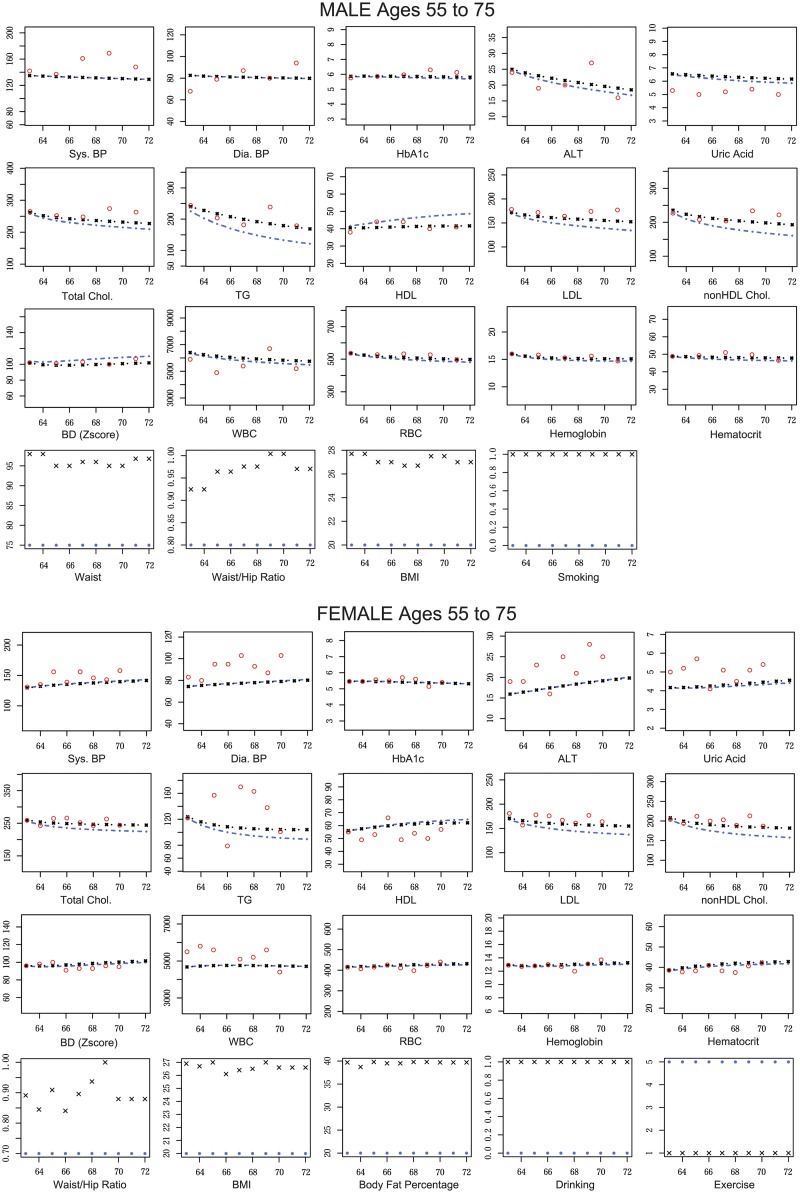
The comparison among predicted and actual blood test values of representative unhealthy male and female. The predicted values are calculated both under actual and healthy conditions for representative unhealthy male and female. The predicted blood test values are calculated by our estimated state space model and ***z***_*k*,*t*_ (actual and healthy) without filtering steps. Red circles are observed blood test values, black lines are predicted blood test values under actual condition, and blue lines are predicted blood test values under the unhealthy condition. Black crossed marks are actual ***z***_*k*,*t*_, and blue circles are postulated ***z***_*k*,*t*_ under the healthy condition. The horizontal and vertical axes indicate the ages of the selected participant at the medical check-up years and the blood test values, respectively. Thus, the youngest age in the horizontal axis means the participant’s age at the first year of visit to the medical check-up event. Note that, in the calculation, we consider the observation at the first year of visit as ***y***_*k*,1_.

Next, we extracted unhealthy male and female and calculated their predicted blood test values, and (ii-a) actual ***z***_*k*,*t*_ and (ii-b) postulated ***z***_*k*,*t*_ to conduct them to be healthy persons. Here, we consider healthy conditions as AC = 75[cm], W/H-ratio = 0.80, BMI = 20, and non-smoking status for male and W/H-ratio = 0.70, BMI = 20, BFP = 20, non-smoking, non-drinking and exercise every day. The simulation results are illustrated in Fig 4.

In these experiments, when healthy male participant was changed his lifestyle to unhealthy one; AC, W/H-ratio, and BMI, were changed from less than 75, 0.80, and 20, to 95, 1.05, and 30, respectively. Also, their means in Male participants are approximately 85, 0.9, and 24, respectively. Because each element of ***z***_*k*,*t*_ is normalized to mean 0 and variance 1 in the application, the rate of change of the effect by the *j*th lifestyle (*g*_*i*,*j*_ × *z*_*k*,*t*,*j*_) to the *i*th hidden variable in this case is almost −1, where *g*_*i*,*j*_ is the *i*th row and the *j*th column element of *G* and *z*_*k*,*t*,*j*_ is the *j*th element of ***z***_*k*,*t*_. On the other hand, smoking and drinking were changed from 0 to 1. The same applies to the opposite case. Similar to the previous experiments, Dyslipidemia Group seems to be the most affected blood test values. In these experiments, the male under unhealthy condition gradually decreases TG and LDL and finally becomes healthy from Dyslipidemia when he is 72 years old. In contrast to the previous experiments, ALT, Uric Acid, HbA1c, BDs, WBC, RBC, Hemoglobin, and Hematocrit are weakly affected both in the Male and Female. In both persons, BPs and HbA1c are not affected.

### Genetic effect analysis

Finally, we analyzed the prediction errors among the predictive and observed values (*observed* *value*—*predicted* *value*). In some participants, there is a clear discrepancy and we assume that they are due to unobserved or unused information such as genetic effects. In the Hirosaki cohort study, genetic data was also obtained in 2014 and 2015, and we focused on detecting associated SNPs for these discrepancies.

**Fig 5:**
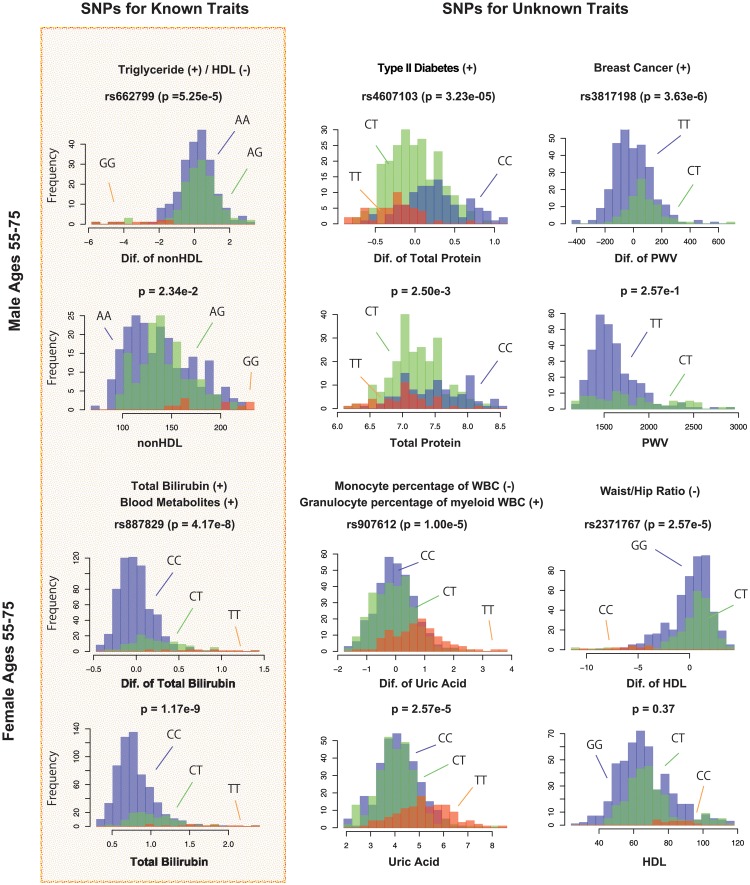
Histograms of the observed blood test values and prediction errors among predicted blood test values of our proposed models and the observed blood test values. The first and the second rows display the results for the Male and the third and the fourth rows display the results for the Female. The left histograms indicate the association of known SNPs and known traits. The middle and right histograms indicate the association of known SNPs and unknown traits. The sign on the right of the blood tests indicates the positive (+) and negative (-) effects from SNPs. Blue, green, and red histograms show the cases of major homo, hetero, and minor risk alleles, respectively.

Thus, we firstly created a list of known SNPs from GWAS catalog [18] by indexing names of the used 38 blood tests as queries and obtained 3, 924 SNPs, of which 915 SNPs are included in our SNP array. We then (i) performed normalization transformation [19] to the observation values and the prediction errors and (ii) checked the genetic associations of these SNPs with all 38 traits by statistical tests (linear regression analysis under additive, recessive, and dominant models with adjustment of age and BMI). To extract firm associations, we set the adjusted threshold for *p* value lower than 0.05/915 = 5.46E − 5 by the Bonferroni method. Note that, in this analysis, some observed values can be generated by the same participants diagnosed at different years and, in these cases, we used mean values to avoid the inflation of *p*-values. Consequently, we obtained SNPs that were strongly associated to the prediction errors in both cases. Parts of the results are illustrated in Fig 5.

It is known that rs662799 is associated with higher TG and cholesterol levels [20] and participants who have this SNP were predicted to exhibit positive prediction errors for nonHDL cholesterol levels (*p* = 5.25E − 5) in the male result. Additionally, rs887829 is popularly associated with total bilirubin levels [21] and participants who have this SNP were predicted to exhibit positive prediction errors for total bilirubin levels (*p* = 4.17E − 8) in the female result. However, in contrast to the results of rs887829, the strong association of rs662799 with nonHDL cholesterol levels could not be detected when using the observation values (*p* = 2.34E − 2). In the middle and right of this figure, novel effects of SNPs were suggested in the associations of the prediction errors. rs4607103 and rs3817198 are known to have an association with type II Diabetes [22] and Breast cancer [23], respectively, but associations with total protein levels (*p* = 3.23E − 5) and PWV (*p* = 3.63E − 5) were indicated in the male results, respectively. Similarly, rs907612 and rs2371767 were indicated to have associations with Uric Acid levels (*p* = 1.00E − 5) and HDL cholesterol levels (*p* = 2.57E − 5) in the female results, respectively, but these SNPs are known to have associations with monocyte and granulocyte [24] and Waist/Hip-ratio [25], respectively. Among them, strong associations of rs4607103, rs3817198 and rs2371767 could not be detected when using observation values.

## Discussion and conclusion

Generally, we could control some blood test values in response to the changes of body composition values, lifestyles, and social status. In this study, we focus on chronic diseases such as Diabetes and high blood pressure and selected 38 blood test values listed in Table 1. Then, we defined ***z***_*k*,*t*_ as clinical target values to be controlled as listed in Table 2 and clarified their effect to the blood test values. Body composition values are considered as endpoints of both exercise and eating habits because it is difficult to directly clarify eating habits. Then, we assume that the changes in body composition values could partially reflect the effects of eating habits. Actually, other blood tests, *e.g*., *γ*GT and C-reactive protein, could be effective items to investigate chronic diseases, but they have been collected recently in this cohort. On the other hand, it may be interesting to analyze blood test values such as vitamins and hormones for other diseases.

From Fig 1, we can see that many blood test values are highly correlated among them, as well as correlated to body composition values, lifestyles, and social status. In contrast, focusing on their year-differences ***y***_*k*,*t*_ − ***y***_*k*,*t*−1_ and ***z***_*k*,*t*_ − ***z***_*k*,*t*−1_, absolute correlation values are lower than the correlations among ***y***_*k*,*t*_ and ***z***_*k*,*t*_. This indicates that blood test values are not suddenly changed according to the changes of other values (except for the case where they indicate almost the same vital condition, such as in HbA1c and glucose). Thus, we can assume that lifestyle changes gradually impact blood test values.

From the results of estimated matrices *H* and *G* in the proposed SSM, similar to the results obtained in Fig 1, the blood test values are clustered into categories such as RBC and Dyslipidemia Groups. They are regulated by the same hidden variables ***x***_*k*,*t*_ and environmental factors ***z***_*k*,*t*_ and assumed to be similarly varied. Additionally, we can see that body composition values can control some indicators for chronic diseases, *e.g*., Dyslipidemia Groups, but simultaneously and oppositely regulate RBC Groups. It is quite natural that chemical compounds are mutually regulated or share the same chemical pathways and they should be simultaneously affected even if we change only a part of the blood test values.

Then, we postulated the case of healthy or unhealthy persons suddenly changing their body composition values and lifestyles and predicted time-transitions of their blood test values. In both males and females, indicators for Dyslipidemia such as total cholesterol, LDL, HDL, and TG, are strongly affected by changes in body composition values such as AC, W/H-ratio, and BMI. These results are intuitively natural because serum cholesterol levels show the lipid profiles in blood whereas body composition values show subcutaneous and visceral fat. Thus, the amount of cholesterols should have a mutual relationship with the body composition values. On the other hand, HbA1c and BPs are slightly affected by the changes of body composition values and lifestyle changes. Exercise could be one of several factors related to and prevent diabetes; however, even if you exercise, you still may get diabetes due to other factors. For example, HbA1c levels can increase if the participants started exercising but were still eating and drinking too much. As a result, their clear relationship cannot be detected in the experiments and we consider these values could be gradually changed over time based on their entire lifestyle. In contrast, ALT, Uric Acid, and BDs values are varied only in males in relation to the changes of indicators for Dyslipidemia. We assume that these blood test values are likely to be changed in males and these values are stable enough in females to reveal the change of trends in our dataset. Consequently, we conclude that the body composition values and lifestyle changes can be a clinical target of Dyslipidemia and the risk of the disease might be controlled by keeping them within regular ranges. Our quantitative results using the mathematical simulation are consistent with types of intuitively reasonable knowledge and capable of updating them in more detail. For other blood tests that are targets of other diseases, such as Diabetes, we have to add other controllable targets in environmental factors. For example, time of eating dinner and composition of the meal can be good factors.

Although the observation data are well predicted for the most part, if predicted values still vary from the observation data in only some participants, they do not share the estimated relationships represented by *A*, *H*, and *G*. In this case, we consider that other factors, which are not included in the model, could affect to their relationships or observation variables. To clarify these factors, finally, we evaluated genetic effects for the correction of prediction errors among the predictive values under the participant’s actual conditions and observed values. We discovered some interesting findings about detected SNPs. In the results of SNPs with known traits, rs662799 has a strong association with higher TG and cholesterol levels [20] and this association could be reproduced when using the prediction errors (*p* = 5.25E − 5). However, a strong association could not be detected when using observation values (*p* = 2.34E − 2). In the results of SNPs with novel traits, the association of rs907612 with uric acid levels was detected by using either prediction errors or observation values. However, associations of rs4607103, rs3817198 and rs2371767 with total protein, PWV and HDL cholesterol, respectively, were detected only by using prediction errors. rs4607103 was suggested to be associated with type II Diabetes [22] and Diabetes is also known to be associated with the increase of total protein. Similarly, rs3817198 had an association with breast cancer [23] and recent studies indicated the association of breast cancer and PWV [26, 27]. The association of rs2371767 to waist/hip-ratio was previously indicated [25] and we can assume that the waist/hip-ratio has an association with the levels of HDL cholesterol because both values can be used as indicators for metabolic syndrome. Thus, these associations seem to be novel findings of genetic effects. Moreover, similar to our experiments, rs907612 and rs2371767 were recently obtained in previous studies [24, 25] by conditioning several covariates, *e.g*., age, exercise, smoking history and drinking history, using a large number of participants (both studies utilized approximately 200, 000 participants). Such approaches could clarify the minute effects of SNPs that could not be detected using traditional genome wide association studies, which condition a few covariates or principal components and capture SNPs with higher effect size. Because our approach can utilize time-series information for detecting associated SNPs, it could especially enhance the detection power in contrast to the traditional non-time series approaches.

The extension of the model to include the presence of SNPs enables us to evaluate their effect to the blood test values and to predict them more accurately. However, such extension makes the calculation heavier depending on the dimension of ***z***_*k*,*t*_ and it is difficult to include all obtained SNPs in the proposed model. Thus, extended models or more efficient algorithm might be required to include SNPs effects. For example, the calculation using linear mixed models are computationally efficient and it can be applied with designing appropriate covariance structure for time-series data. In addition, since random effects could correct for population structure and family relatedness also in SSM, it can be extensions of the proposed model. Some other factors can also affect to the changes of blood test values. The analysis including SNP effects, random effects, and other factors is one of future works. Our proposed model can express linear relationships under the Gaussian assumption among blood test values and environmental factors, but many natural processes, *e.g*., chemical reaction networks and biological signal processing, are generally represented as nonlinear structures. Moreover, the observation noises of some blood test values could not depend on the Gaussian noises, for example, TG levels can suddenly increase due to eating habits before medical check-up. The use of a nonlinear structure and non-Gaussian assumption has the potential to clarify more detailed relationship among them.

## Supporting information

S1 Appendix A solution to infer parameter values of the proposed model. The detailed solution of estimating parameter values using the EM-algorithm for the proposed model.
(PDF)

S1 Fig Histogram of ages and blood test values of included and excluded participants. The histograms of ages and 38 observation values ***y***_*k*,*t*_ of included and excluded samples for both dataset.
(PDF)

S2 Fig The lowest BIC score of each system dimension *p*. The lowest BIC score of each system dimension *p* (*p* = 1, …, 15) for both dataset.
(PDF)

S3 Fig A graphical view of regulatory matrix *F*. The regulatory relationships among ***x***_*k*,*t*_ and ***z***_*k*,*t*_ generated from *F* for both dataset.
(PDF)
