# Supplementary material for: Prediction of blood test values under different lifestyle scenarios using time-series electronic health record
Source: PLoS One. 2020 Mar 20;15(3):e0230172. doi: 10.1371/journal.pone.0230172 (PMC7083324; doi:10.1371/journal.pone.0230172)
Supplement: S1 Appendix — The detailed solution of estimating parameter values using the EM-algorithm for the proposed model. (PDF) [file pone.0230172.s001.pdf]

S1 Appendix

Kalman Filter Algorithm

For the calculation of the conditional distribution of the hidden variables $\boldsymbol{x}_{k,t|t-1}$, the prediction, filtering, and smoothing of the Kalman filter are performed by the following formulas:

- Prediction:

$$\boldsymbol{x}_{k,t|t-1} = A\boldsymbol{x}_{k,t-1|t-1} + G\boldsymbol{z}_{k,t}, \tag{S-1}$$
$$\Sigma_{k,t|t-1} = A\Sigma_{k,t-1|t-1}A' + Q, \tag{S-2}$$

- Filtering:

$$\boldsymbol{x}_{k,t|t} = \boldsymbol{x}_{k,t|t-1} + \Sigma_{k,t|t}R^{-1}(\boldsymbol{y}_{k,t} - \boldsymbol{x}_{k,t|t-1}), \tag{S-3}$$
$$\Sigma_{k,t|t} = (R^{-1} + \Sigma_{k,t|t-1}^{-1})^{-1}, \tag{S-4}$$

- Smoothing

$$\boldsymbol{x}_{k,t|T} = \boldsymbol{x}_{k,t|t} + J_{k,t}(\boldsymbol{x}_{k,t+1|T} - \boldsymbol{x}_{k,t+1|t}), \tag{S-5}$$
$$\Sigma_{k,t|T} = \Sigma_{k,t|t} + J_{k,t}(\Sigma_{k,t+1|T} - \Sigma_{k,t+1|t})J'_{k,t}, \tag{S-6}$$
$$\Sigma_{k,t,t-1|T} = \Sigma_{k,t|t}J'_{k,t-1} + J_{k,t}(\Sigma_{k,t+1,t|T} - A\Sigma_{k,t|t})J'_{k,t-1}, \tag{S-7}$$
$$J_{k,t} = \Sigma_{k,t|t}A'\Sigma_{k,t+1|t}^{-1}, \tag{S-8}$$

$$\Sigma_{k,T,T-1|T} = (I - \Sigma_{k,T|T}R^{-1})A\Sigma_{k,T-1|T-1}, \tag{S-9}$$

where Exp$[\boldsymbol{x}_{k,t}]$ given $\boldsymbol{y}_{k,1}, \ldots, \boldsymbol{y}_{k,s}$ is represented by $\boldsymbol{x}_{k,t|s}$, Var$[\boldsymbol{x}_{k,t}]$ given $\boldsymbol{y}_{k,1}, \ldots, \boldsymbol{y}_{k,s}$ is represented by $\boldsymbol{\Sigma}_{k,t|s}$, and the covariance between $\boldsymbol{x}_{k,t}$ and $\boldsymbol{x}_{k,t-1}$ given $\boldsymbol{y}_{k,1}, \ldots, \boldsymbol{y}_{k,s}$ is represented by $\boldsymbol{\Sigma}_{k,t,t-1|s}$. They are used in the Expectation-step of the Expectation-Maximization (EM) algorithm.

The EM-algorithm for $L1$-regularized SSM

In the Expectation-step, the conditional expectation of the joint log-likelihood of the complete data set $q(\boldsymbol{\theta}|\boldsymbol{\theta}_l)$ is calculated by

$$\begin{aligned} q(\boldsymbol{\theta}|\boldsymbol{\theta}_l) &= \mathrm{Exp}[\log P(X,Y;\boldsymbol{\theta})|Y;\boldsymbol{\theta}_l] \\ &= -\frac{K}{2}\log|\Sigma_0| - \frac{1}{2}\sum_{k=1}^{K} T_k \log|Q| - \frac{1}{2}\sum_{k=1}^{K} T_k \log|R| - \frac{1}{2}\mathrm{tr}[\sum_{k=1}^{K}\Sigma_{k,0|T_k}^{-1}\{(\boldsymbol{x}_{k,0|T_k} - \boldsymbol{\mu}_{k,0})(\boldsymbol{x}_{k,0|T_k} - \boldsymbol{\mu}_{k,0})' + \Sigma_{k,0|T_k}\}] \\ &\quad -\frac{1}{2}\mathrm{tr}\{Q^{-1}(V_t - V_{lag}F' - FV'_{lag} + FV_{t-1}F' + FE_{t-1}G' + GE'_{t-1}F' - E_{lag}G' - GE_{lag} + GZG')\} \\ &\quad -\frac{1}{2}\mathrm{tr}[R^{-1}\sum_{k=1}^{K}\sum_{t=1}^{T_k}\{(\boldsymbol{y}_{k,t} - H\boldsymbol{x}_{k,t|T_k})(\boldsymbol{y}_{k,t} - H\boldsymbol{x}_{k,t|T_k})' + H\Sigma'_{k,t|T_k}H'\}] \\ &\quad -N(\sum_{k=1}^{K} T_k + \frac{1}{2})\log 2\pi - \sum_{i=1}^{p}\sum_{j=1}^{p}\lambda_i^{(s)}|A_{i,j}| - \sum_{i=1}^{p}\sum_{j=1}^{m}\lambda_i^{(s)}|G_{i,j}| - \sum_{i=1}^{q}\sum_{j=1}^{p}\lambda_i^{(o)}|H_{i,j}|, \end{aligned} \tag{S-10}$$

where

$$V_t = \sum_{k=1}^{K}\sum_{t\in\mathcal{T}_k}(\Sigma_{k,t|T_k} + \boldsymbol{x}_{k,t|T_k}\boldsymbol{x}'_{k,t|T_k}), \tag{S-11}$$

$$V_{lag} = \sum_{k=1}^{K}\sum_{t\in\mathcal{T}_k}(\Sigma_{k,t,t-1|T_k} + \boldsymbol{x}_{k,t|T_k}\boldsymbol{x}'_{k,t-1|T_k}), \tag{S-12}$$

$$V_{t-1} = \sum_{k=1}^{K}\sum_{t\in\mathcal{T}_k}(\Sigma_{k,t-1|T_k} + \boldsymbol{x}_{k,t-1|T_k}\boldsymbol{x}'_{k,t-1|T_k}), \tag{S-13}$$

$$W_t = \sum_{k=1}^{K} \sum_{t \in \mathcal{T}_k} (\boldsymbol{x}'_{k,t|T_k} \boldsymbol{y}_{k,t}), \tag{S-14}$$

$$\boldsymbol{s}_t = \sum_{k=1}^{K} \sum_{t \in \mathcal{T}_k} \boldsymbol{x}_{k,t|T_k}, \tag{S-15}$$

$$\boldsymbol{s}_{t-1} = \sum_{k=1}^{K} \sum_{t \in \mathcal{T}_k} \boldsymbol{x}_{k,t-1|T_k}, \tag{S-16}$$

$$E_{lag} = \sum_{k=1}^{K} \sum_{t \in \mathcal{T}_k} \boldsymbol{x}_{k,t|T_k} \boldsymbol{z}'_{k,t-1|T_k}, \tag{S-17}$$

$$E_{t-1} = \sum_{k=1}^{K} \sum_{t \in \mathcal{T}_k} \boldsymbol{x}_{k,t-1|T_k} \boldsymbol{z}'_{k,t-1|T_k}, \tag{S-18}$$

$$\boldsymbol{z} = \sum_{k=1}^{K} \sum_{t \in \mathcal{T}_k} \boldsymbol{z}_{k,t-1|T_k}, \tag{S-19}$$

$$Z = \sum_{k=1}^{K} \sum_{t \in \mathcal{T}_k} \boldsymbol{z}_{k,t-1|T_k} \boldsymbol{z}'_{k,t-1|T_k}. \tag{S-20}$$

In the Maximization-step, $\boldsymbol{\theta}_l$ is updated to $\boldsymbol{\theta}_{l+1}$ to be $\boldsymbol{\theta}_{l+1} = \arg\max_{\boldsymbol{\theta}} q(\boldsymbol{\theta}|\boldsymbol{\theta}_l)$. Let $\boldsymbol{v}_{t,i}$, $\boldsymbol{v}_{lag,i}$, $\boldsymbol{v}_{t-1,i}$, $\boldsymbol{e}_{t,i}$ and $\boldsymbol{e}_{lag,i}$ set a transpose of ith row vector of V_t, V_{lag}, V_{t-1}, E_{lag} and E_{t-1}, respectively. Further, set $s_{t,i}$ and $s_{t-1,i}$ as an ith element of $\boldsymbol{s}_t$ and $\boldsymbol{s}_{t-1}$, and $v_{t,i,j}$ and $v_{t-1,i,j}$ as an ith row jth column element of V_t and V_{t-1}, respectively. Then, $\boldsymbol{\theta}$ is updated as

$$\boldsymbol{a}_i = \arg\min_{\boldsymbol{a}_i} \{\boldsymbol{a}'_i V_{t-1} \boldsymbol{a}_i + 2\boldsymbol{v}'_{t-1,i}\boldsymbol{a}_i - 2\boldsymbol{v}'_{lag,i}\boldsymbol{a}_i + 2\boldsymbol{g}'_i E'_{t-1}\boldsymbol{a}_i + 2q_i \sum_{j=1}^{p} \lambda_i^{(s)} |a_{i,j}|\}, \tag{S-21}$$

$$s.t. \quad \boldsymbol{a}_i^{\mathcal{A}_i} = V_{t-1}^{\mathcal{A}_i^{-1}} (\boldsymbol{v}_{lag,i}^{\mathcal{A}_i} - \boldsymbol{v}_{t-1,i}^{\mathcal{A}_i} - u_i \boldsymbol{s}_{t-1}^{\mathcal{A}_i} - E_{t-1}^{\mathcal{A}_i} \boldsymbol{g}_i^{\mathcal{A}_i} - q_i \lambda_i^{(s)} sign(\boldsymbol{a}_i^{\mathcal{A}_i})), \tag{S-22}$$

$$\boldsymbol{g}_i = \arg\min_{\boldsymbol{g}_i} \{\boldsymbol{g}'_i Z \boldsymbol{g}_i + 2\boldsymbol{f}'_i E_{t-1} \boldsymbol{g}_i - 2\boldsymbol{e}'_{lag,i} \boldsymbol{g}_i + 2q_i \sum_{j=1}^{m} \lambda_i^{(s)} |g_{i,j}|\}, \tag{S-23}$$

$$s.t. \quad \boldsymbol{g}_i^{\mathcal{G}_i} = Z^{\mathcal{G}_i^{-1}} (\boldsymbol{e}_{lag,i}^{\mathcal{G}_i} - E_{t-1}^{\mathcal{G}'_i} \boldsymbol{f}_i^{\mathcal{G}_i} - u_i \boldsymbol{z}^{\mathcal{G}_i} - q_i \lambda_i^{(s)} sign(\boldsymbol{g}_i^{\mathcal{G}_i})), \tag{S-24}$$

$$\boldsymbol{h}_i = \arg\min_{\boldsymbol{h}_i} \{\boldsymbol{h}'_i V_t \boldsymbol{h}_i - 2\boldsymbol{w}'_{t,i} \boldsymbol{h}_i + 2r_i \sum_{j=1}^{p} \lambda_i^{(o)} |h_{i,j}|\}, \tag{S-25}$$

$$s.t. \quad \boldsymbol{h}_i^{\mathcal{H}_i} = V_t^{\mathcal{H}_i^{-1}} (\boldsymbol{w}_{t,i}^{\mathcal{H}_i} - r_i \lambda_i^{(o)} sign(\boldsymbol{h}_i^{\mathcal{H}_i})), \tag{S-26}$$

$$Q = \frac{1}{\sum_{k=1}^{K} T_k} (V_t - V_{lag}F' - FV'_{lag} + FV_tF' + FE_{lag}G' + GE'_{lag}F' - E_tG' - GE_t + GZG'), \tag{S-27}$$

$$R = \frac{1}{\sum_{k=1}^{K} T_k} \sum_{t \in \mathcal{T}_{k,obs}} \{(\boldsymbol{y}_{k,t} - \boldsymbol{x}_{k,t|T_k})(\boldsymbol{y}_{k,t} - \boldsymbol{x}_{k,t|T_k})' + \Sigma_{k,t|T_k}\}, \tag{S-28}$$

$$\boldsymbol{\mu}_{k,0} = \boldsymbol{x}_{k,0|T_k} \quad (k = 1, \ldots, K), \tag{S-29}$$

where $\mathcal{A}_i$, $\mathcal{G}_i$, and $\mathcal{H}_i$ be active sets of elements for $\boldsymbol{a}_i$, $\boldsymbol{g}_i$, and $\boldsymbol{h}_i$, *i.e.*, $\forall\{a_{i,j} \neq 0\} \in \mathcal{A}_i$ for $i = 1, \ldots, p$, $\forall\{g_{i,j} \neq 0\} \in \mathcal{G}_i$ for $i = 1, \ldots, p$, and $\forall\{h_{i,j} \neq 0\} \in \mathcal{H}_i$ for $i = 1, \ldots, q$, respectively, and *sign* means a sign vector consisting positive (+1) or negative (-1) values. The descriptions of $^{\mathcal{A}_i}$, $^{\mathcal{G}_i}$, and $^{\mathcal{H}_i}$ stand for an $|\mathcal{A}_i| \times |\mathcal{A}_i|$ matrix or an $|\mathcal{A}_i|$ dimensional vector, a $|\mathcal{G}_i| \times |\mathcal{G}_i|$ matrix or a $|\mathcal{G}_i|$ dimensional vector, and a $|\mathcal{H}_i| \times |\mathcal{H}_i|$ matrix or an $|\mathcal{H}_i|$ dimensional vector, respectively. We describe $\mathcal{A} = \{\mathcal{A}_1, \ldots, \mathcal{A}_i\}$, $\mathcal{G} = \{\mathcal{G}_1, \ldots, \mathcal{G}_i\}$, and $\mathcal{H} = \{\mathcal{H}_1, \ldots, \mathcal{H}_i\}$.

Here, we have to evaluate all plausible active sets $\mathcal{A}_i$, $\mathcal{G}_i$, and $\mathcal{H}_i$ to obtain Eqs.(S-22)-(S-26). However, through the proposed parameter optimization algorithm, the regularization parameters $\lambda_i^{(s)}$ and $\lambda_i^{(o)}$ are gradually changed

and $\mathcal{A}_i$, $\mathcal{G}_i$, and $\mathcal{H}_i$ are almost the same as the previously obtained active sets. Thus, we only evaluate active sets that are generated from the previously obtained ones by adding and removing at most two elements. For example, if we have $\mathcal{A}_i = \{0, 1, 4\}$ and $p = 4$, we only evaluate $\mathcal{A}_i = \{0\}$, $\{1\}$, $\{4\}$, $\{0, 1\}$, $\{0, 4\}$, $\{1, 4\}$, $\{0, 1, 4\}$, $\{1, 2, 4\}$, $\{1, 3, 4\}$, $\{0, 2, 4\}$, $\{0, 3, 4\}$, $\{0, 1, 2\}$, $\{0, 1, 3\}$, $\{0, 1, 2, 4\}$, $\{0, 1, 3, 4\}$, $\{0, 1, 2, 3, 4\}$. This can dramatically reduce the computational cost with keeping the performance like Least Absolute Shrinkage and Selection Operator (LASSO).

Parameter Optimization Algorithm with $L1$ Regularization

Let $\mathcal{A}_i$, $\mathcal{G}_i$, and $\mathcal{H}_i$ be active sets of elements for $\boldsymbol{a}_i$, $\boldsymbol{g}_i$, and $\boldsymbol{h}_i$, *i.e.*, $\forall\{a_{i,j} \neq 0\} \in \mathcal{A}_i$ for $i = 1, \ldots, p$, $\forall\{g_{i,j} \neq 0\} \in \mathcal{G}_i$ for $i = 1, \ldots, p$, and $\forall\{h_{i,j} \neq 0\} \in \mathcal{H}_i$ for $i = 1, \ldots, q$, respectively. Because of the combination of the regularization terms and a state space representation, updating an element of $\boldsymbol{\lambda}^{(s)} = (\lambda_1^{(s)}, \ldots, \lambda_p^{(s)})'$ and $\boldsymbol{\lambda}^{(o)} = (\lambda_1^{(o)}, \ldots, \lambda_q^{(o)})'$influences the other active sets. Thus, it is difficult to select the optimal active sets of $\mathcal{A}_i$, $\mathcal{G}_i$, and $\mathcal{H}_i$, and the values of $\boldsymbol{\theta}$, $\boldsymbol{\lambda}^{(s)}$, and $\boldsymbol{\lambda}^{(o)}$, at the same time. Therefore, we propose a novel algorithm to separately update them in each row. In this algorithm, we introduce auxiliary sets $\tilde{\mathcal{A}}_i$, $\tilde{\mathcal{G}}_i$, and $\tilde{\mathcal{H}}_i$, and constraint that the active sets $\mathcal{A}_i$, $\mathcal{G}_i$, and $\mathcal{H}_i$ are selected from the auxiliary sets, *i.e.*, $\mathcal{A}_i \subseteqq \tilde{\mathcal{A}}_i$, $\mathcal{G}_i \subseteqq \tilde{\mathcal{G}}_i$, and $\mathcal{H}_i \subseteqq \tilde{\mathcal{H}}_i$.

Algorithm

1. Set $\boldsymbol{\lambda}^{(s)} = \mathbf{0}$ and $\boldsymbol{\lambda}^{(o)} = \mathbf{0}$, and $\tilde{\mathcal{A}}_i$, $\tilde{\mathcal{G}}_i$, and $\tilde{\mathcal{H}}_i$ to be full. Then, recursively update $\boldsymbol{\theta}$ using the EM algorithm until convergence is attained. In this step, active sets $\mathcal{A}_i$ $(i = 1, \ldots, p)$, $\mathcal{G}_i$ $(i = 1, \ldots, p)$, and $\mathcal{H}_i$ $(i = 1, \ldots, q)$ consist of all elements, *i.e.*, A, G, and H become dense matrices, since the regularization terms can be neglected.

2. Set the current iteration L_{cur} to be 0 and the maximum number of iterations to be L_{max}.

3. For $i_{upd} = 1, \ldots, p$

   a). Set $\tilde{\mathcal{A}}_{i_{upd}}$ and $\tilde{\mathcal{G}}_{i_{upd}}$ full and $\lambda_{i_{upd}}^{(s)} = 0$ to allow $\boldsymbol{a}_{i_{upd}}$ and $\boldsymbol{g}_{i_{upd}}$ to become dense vectors. Through the following steps, with fixing $\lambda_i^{(s)}$ $(i \neq i_{upd})$ and $\lambda_i^{(o)}$, $\lambda_{i_{upd}}^{(s)}$ is gradually increased to find an optimum $\lambda_{i_{upd}}^{(s)}$ for which the BIC score is minimized.

   b). Calculate conditional expectations using the Kalman filter.

   c). Update $\mathcal{A}$, $\mathcal{G}$, $\mathcal{H}$, and $\boldsymbol{\theta}$ by the algorithm described in the supplemental materials. Here, $\mathcal{A}_i$, $\mathcal{G}_i$ and $\mathcal{H}_i$ can be constructed from $\tilde{\mathcal{A}}_i$, $\tilde{\mathcal{H}}_i$ and $\tilde{\mathcal{G}}_i$, respectively.

   d). Calculate the BIC score and increase $\lambda_{i_{upd}^{(s)}}$ if the regularized log-likelihood is converged. Then, repeat from step (b) until $\mathcal{A}_{i_{upd}}$ and $\mathcal{G}_{i_{upd}}$ become null matrices.

   e). Set $\{\boldsymbol{\lambda}^{(s)}, \boldsymbol{\lambda}^{(o)}, \mathcal{A}, \mathcal{G}, \mathcal{H}, \boldsymbol{\theta}\}$ as the value with the lowest BIC score obtained through the above described steps. Furthermore, set $\tilde{\mathcal{A}}_{i_{upd}} \leftarrow \mathcal{A}_{i_{upd}}$ and $\tilde{\mathcal{G}}_{i_{upd}} \leftarrow \mathcal{G}_{i_{upd}}$.

4. For $i_{upd} = 1, \ldots, q$ and $\mathcal{H}$, the same procedure as $\mathcal{A}$ and $\mathcal{G}$ in step 3 is proceeded.

5. Set $L_{cur} \rightarrow L_{cur} + 1$ and repeat from step 3 until L_{cur} becomes L_{max}.
