# Supplementary material for: Prediction of blood test values under different lifestyle scenarios using time-series electronic health record
Source: PLoS One. 2020 Mar 20;15(3):e0230172. doi: 10.1371/journal.pone.0230172 (PMC7083324; doi:10.1371/journal.pone.0230172)
Supplement: S1 Fig — The histograms of ages and 38 observation values yk,t of included and excluded samples for both dataset. (PDF) [file pone.0230172.s002.pdf]

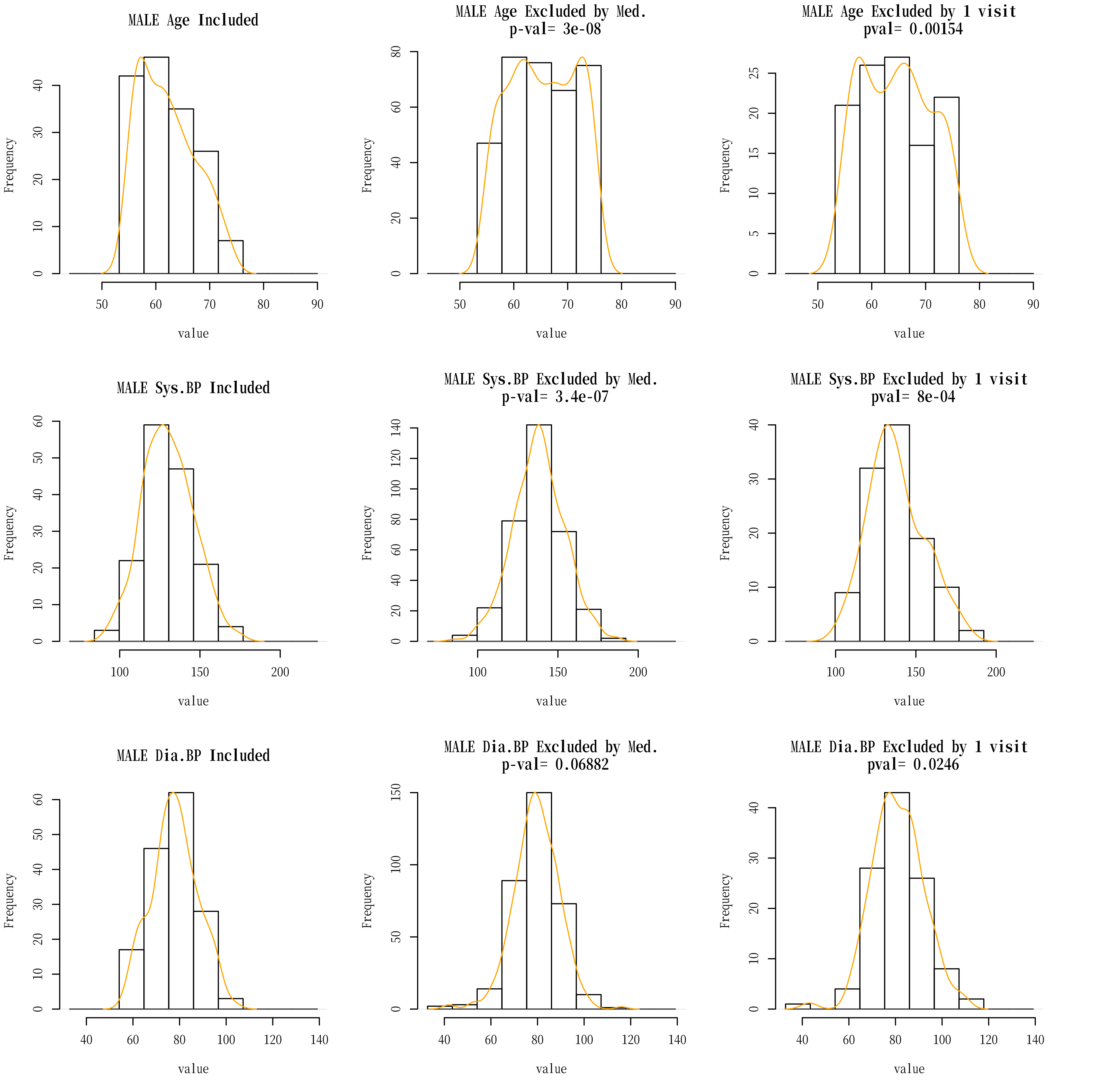
MALE Age Included
MALE Age Excluded by Med.
p-val= 3e-08
MALE Age Excluded by 1 visit
pval= 0.00154
MALE Sys.BP Included
MALE Sys.BP Excluded by Med.
p-val= 3.4e-07
MALE Sys.BP Excluded by 1 visit
pval= 8e-04
MALE Dia.BP Included
MALE Dia.BP Excluded by Med.
p-val= 0.06882
MALE Dia.BP Excluded by 1 visit
pval= 0.0246
Frequency
value

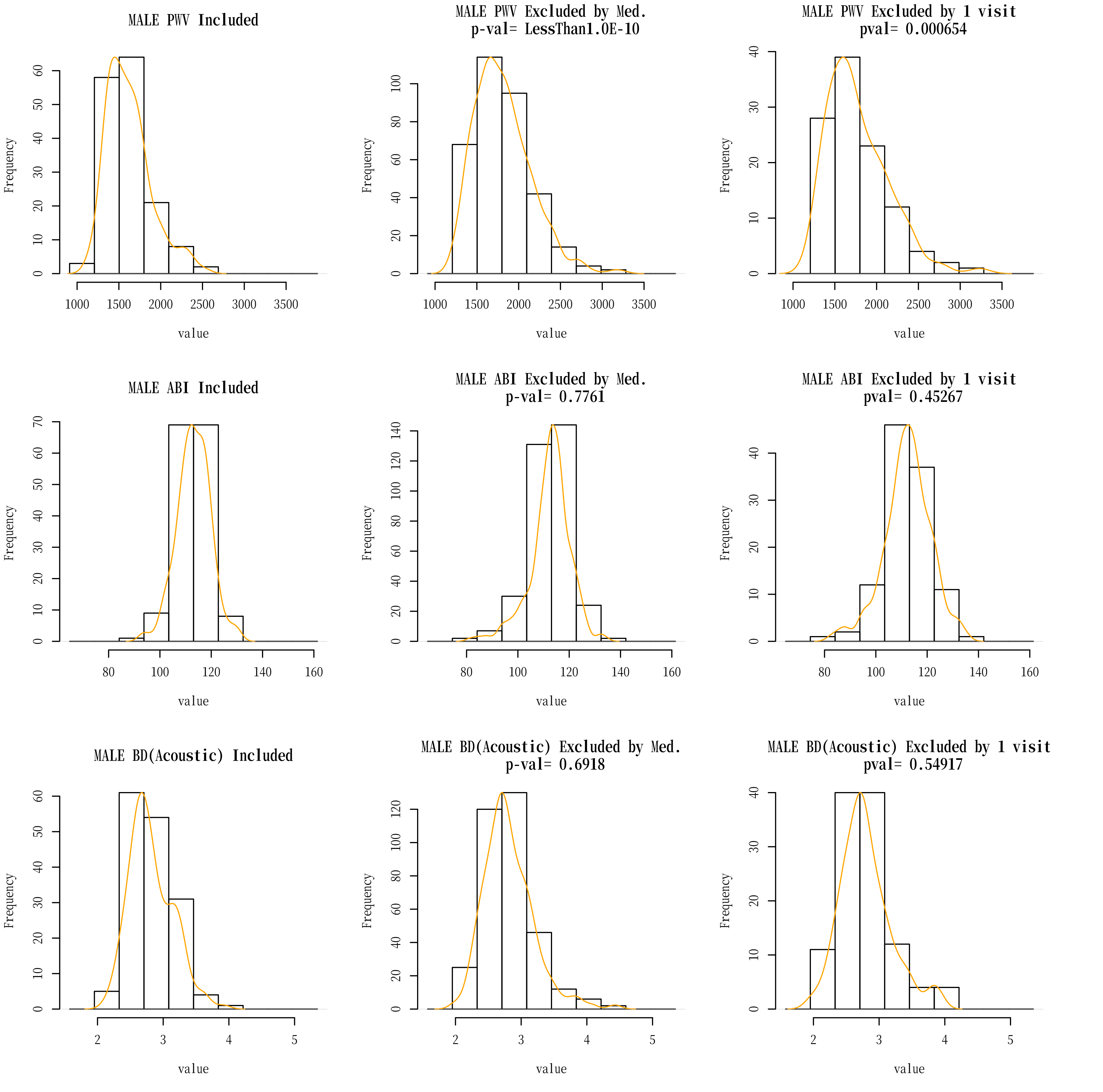
MALE PWV Included
MALE PWV Excluded by Med.
p-val= LessThan1.0E-10
MALE PWV Excluded by 1 visit
pval= 0.000654
MALE ABI Included
MALE ABI Excluded by Med.
p-val= 0.7761
MALE ABI Excluded by 1 visit
pval= 0.45267
MALE BD(Acoustic) Included
MALE BD(Acoustic) Excluded by Med.
p-val= 0.6918
MALE BD(Acoustic) Excluded by 1 visit
pval= 0.54917
Frequency
value
1000
1500
2000
2500
3000
3500
80
100
120
140
160
2
3
4
5

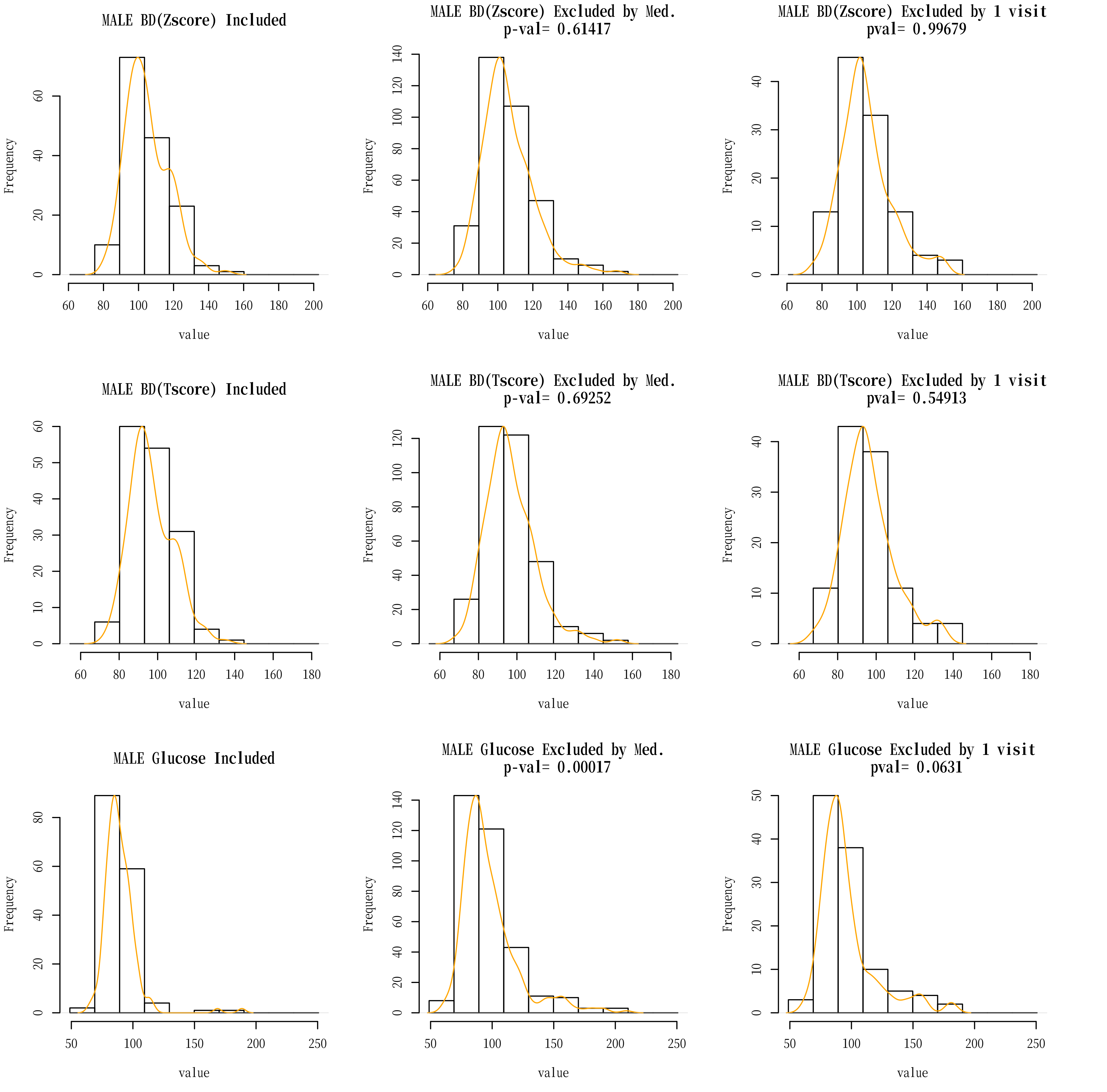
MALE BD(Zscore) Included
Frequency
value
MALE BD(Zscore) Excluded by Med.
p-val= 0.61417
Frequency
value
MALE BD(Zscore) Excluded by 1 visit
pval= 0.99679
Frequency
value
MALE BD(Tscore) Included
Frequency
value
MALE BD(Tscore) Excluded by Med.
p-val= 0.69252
Frequency
value
MALE BD(Tscore) Excluded by 1 visit
pval= 0.54913
Frequency
value
MALE Glucose Included
Frequency
value
MALE Glucose Excluded by Med.
p-val= 0.00017
Frequency
value
MALE Glucose Excluded by 1 visit
pval= 0.0631
Frequency
value

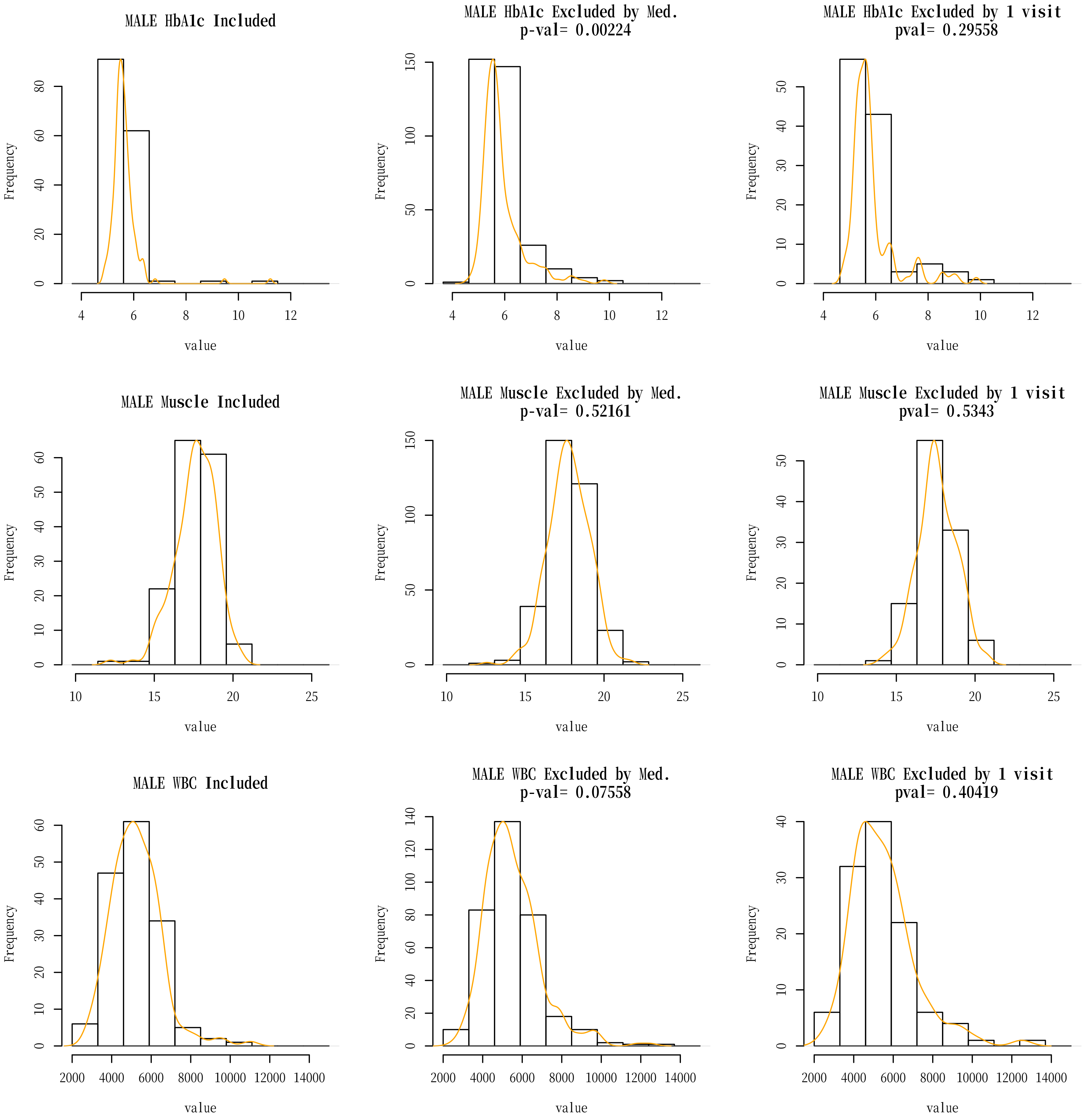
MALE HbA1c Included
MALE HbA1c Excluded by Med.
p-val= 0.00224
MALE HbA1c Excluded by 1 visit
pval= 0.29558
MALE Muscle Included
MALE Muscle Excluded by Med.
p-val= 0.52161
MALE Muscle Excluded by 1 visit
pval= 0.5343
MALE WBC Included
MALE WBC Excluded by Med.
p-val= 0.07558
MALE WBC Excluded by 1 visit
pval= 0.40419
Frequency
value

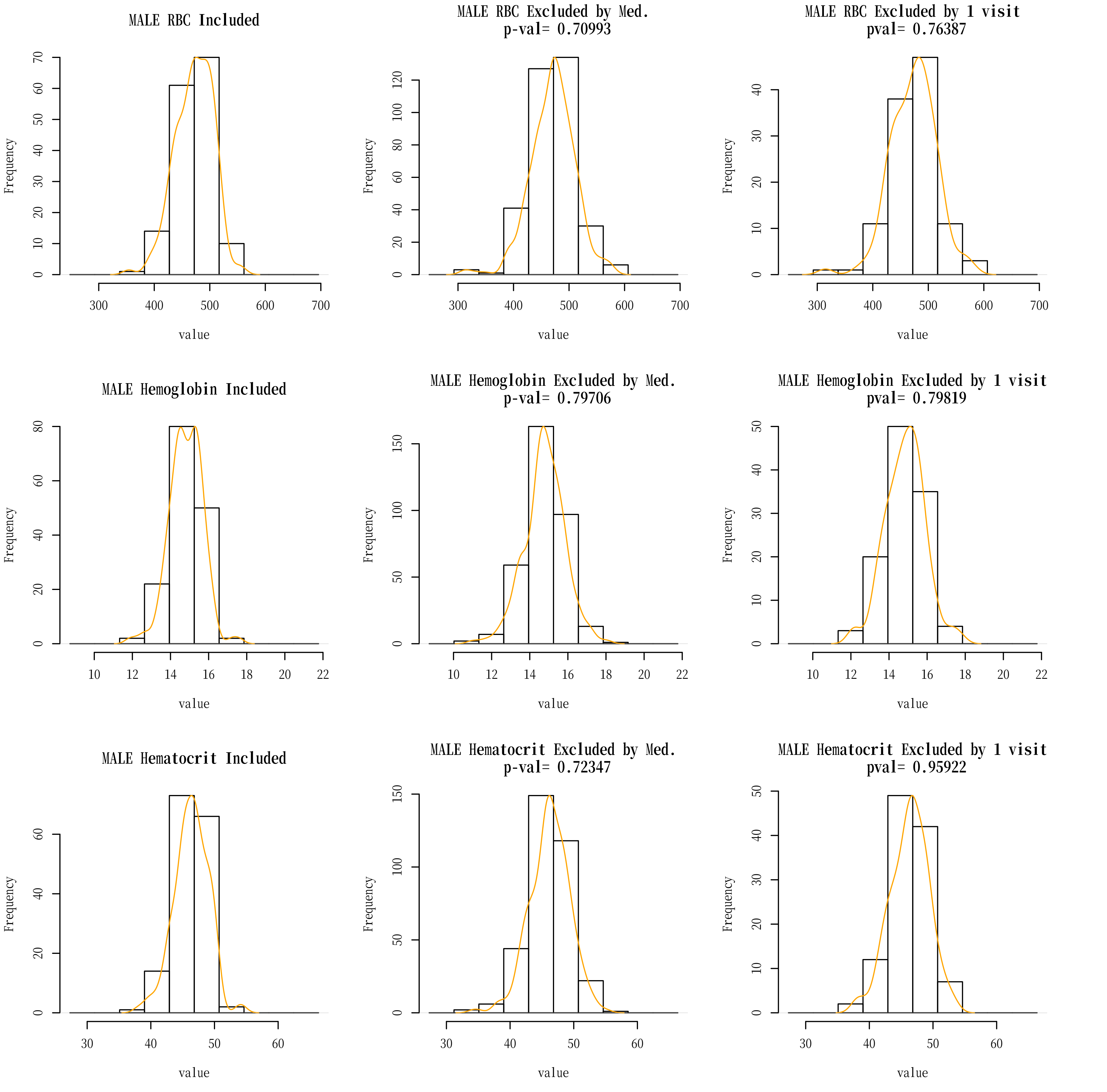
MALE RBC Included
MALE RBC Excluded by Med.
p-val= 0.70993
MALE RBC Excluded by 1 visit
pval= 0.76387
MALE Hemoglobin Included
MALE Hemoglobin Excluded by Med.
p-val= 0.79706
MALE Hemoglobin Excluded by 1 visit
pval= 0.79819
MALE Hematocrit Included
MALE Hematocrit Excluded by Med.
p-val= 0.72347
MALE Hematocrit Excluded by 1 visit
pval= 0.95922
Frequency
value

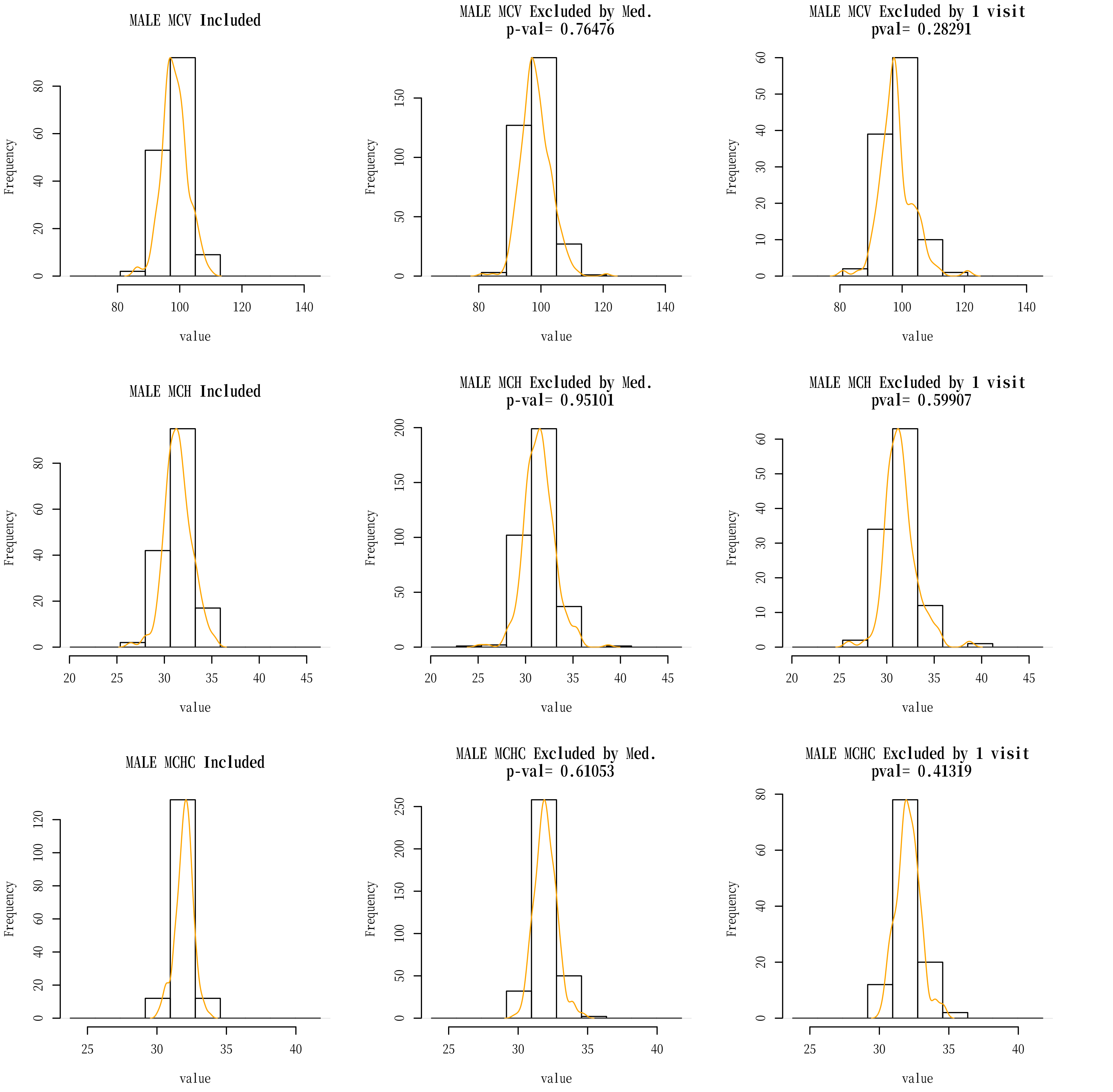
MALE MCV Included
MALE MCV Excluded by Med.
p-val= 0.76476
MALE MCV Excluded by 1 visit
pval= 0.28291
MALE MCH Included
MALE MCH Excluded by Med.
p-val= 0.95101
MALE MCH Excluded by 1 visit
pval= 0.59907
MALE MCHC Included
MALE MCHC Excluded by Med.
p-val= 0.61053
MALE MCHC Excluded by 1 visit
pval= 0.41319
Frequency
value

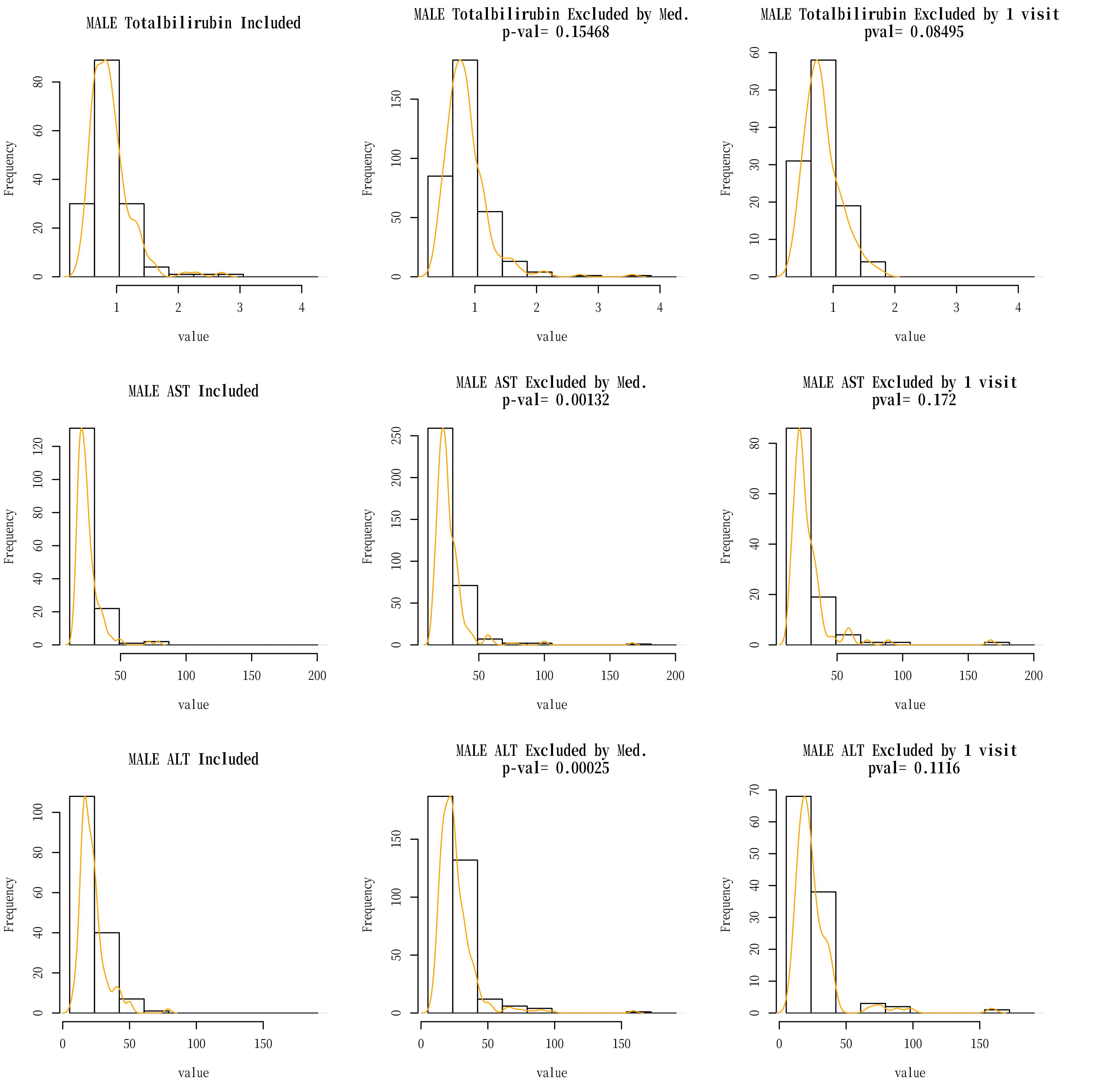
MALE Totalbilirubin Included
MALE Totalbilirubin Excluded by Med.
p-val= 0.15468
MALE Totalbilirubin Excluded by 1 visit
pval= 0.08495
MALE AST Included
MALE AST Excluded by Med.
p-val= 0.00132
MALE AST Excluded by 1 visit
pval= 0.172
MALE ALT Included
MALE ALT Excluded by Med.
p-val= 0.00025
MALE ALT Excluded by 1 visit
pval= 0.1116
Frequency
value

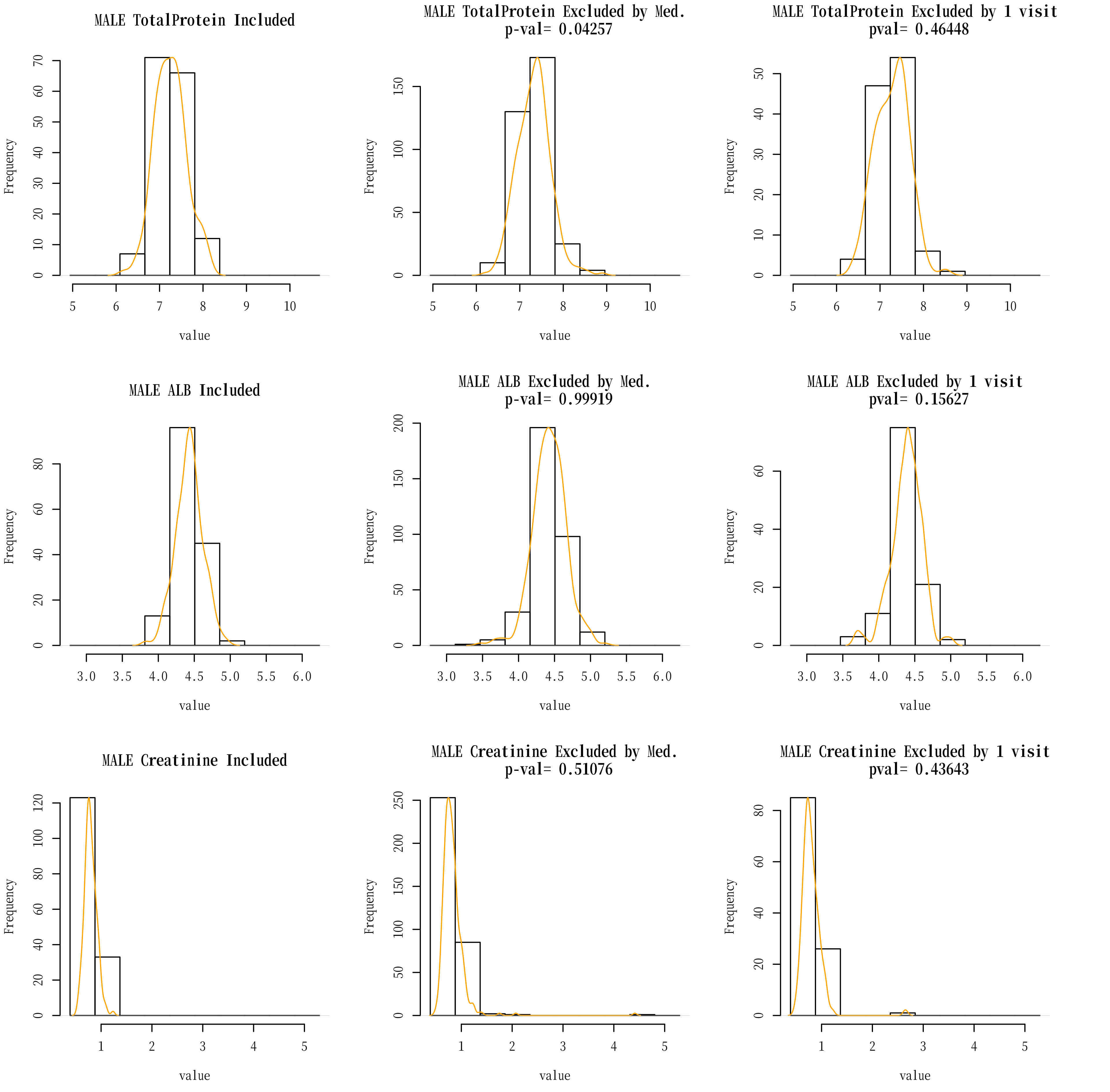
MALE TotalProtein Included
Frequency
value
MALE TotalProtein Excluded by Med.
p-val= 0.04257
Frequency
value
MALE TotalProtein Excluded by 1 visit
pval= 0.46448
Frequency
value
MALE ALB Included
Frequency
value
MALE ALB Excluded by Med.
p-val= 0.99919
Frequency
value
MALE ALB Excluded by 1 visit
pval= 0.15627
Frequency
value
MALE Creatinine Included
Frequency
value
MALE Creatinine Excluded by Med.
p-val= 0.51076
Frequency
value
MALE Creatinine Excluded by 1 visit
pval= 0.43643
Frequency
value

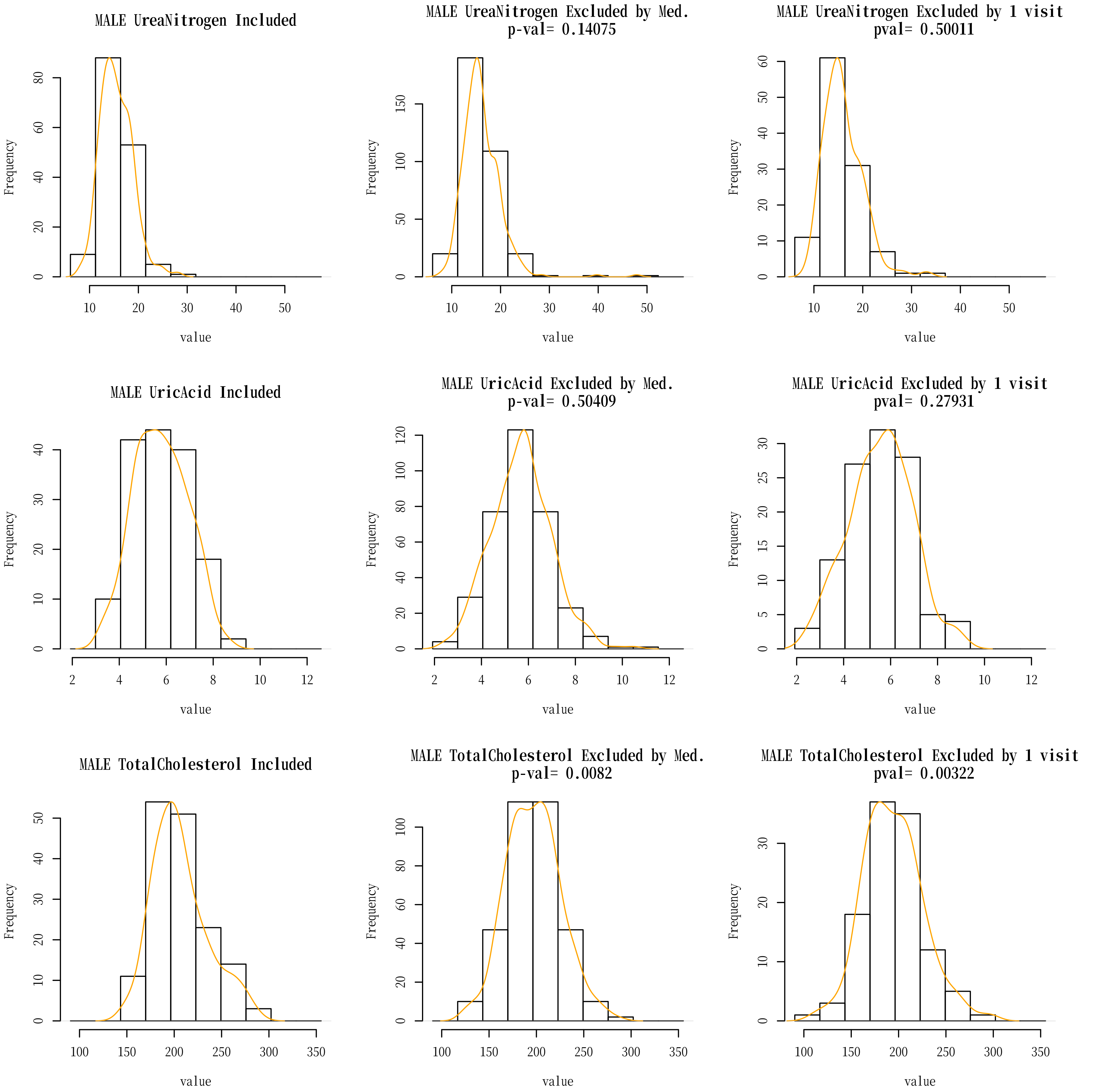
MALE UreaNitrogen Included
MALE UreaNitrogen Excluded by Med.
p-val= 0.14075
MALE UreaNitrogen Excluded by 1 visit
pval= 0.50011
MALE UricAcid Included
MALE UricAcid Excluded by Med.
p-val= 0.50409
MALE UricAcid Excluded by 1 visit
pval= 0.27931
MALE TotalCholesterol Included
MALE TotalCholesterol Excluded by Med.
p-val= 0.0082
MALE TotalCholesterol Excluded by 1 visit
pval= 0.00322
Frequency
value

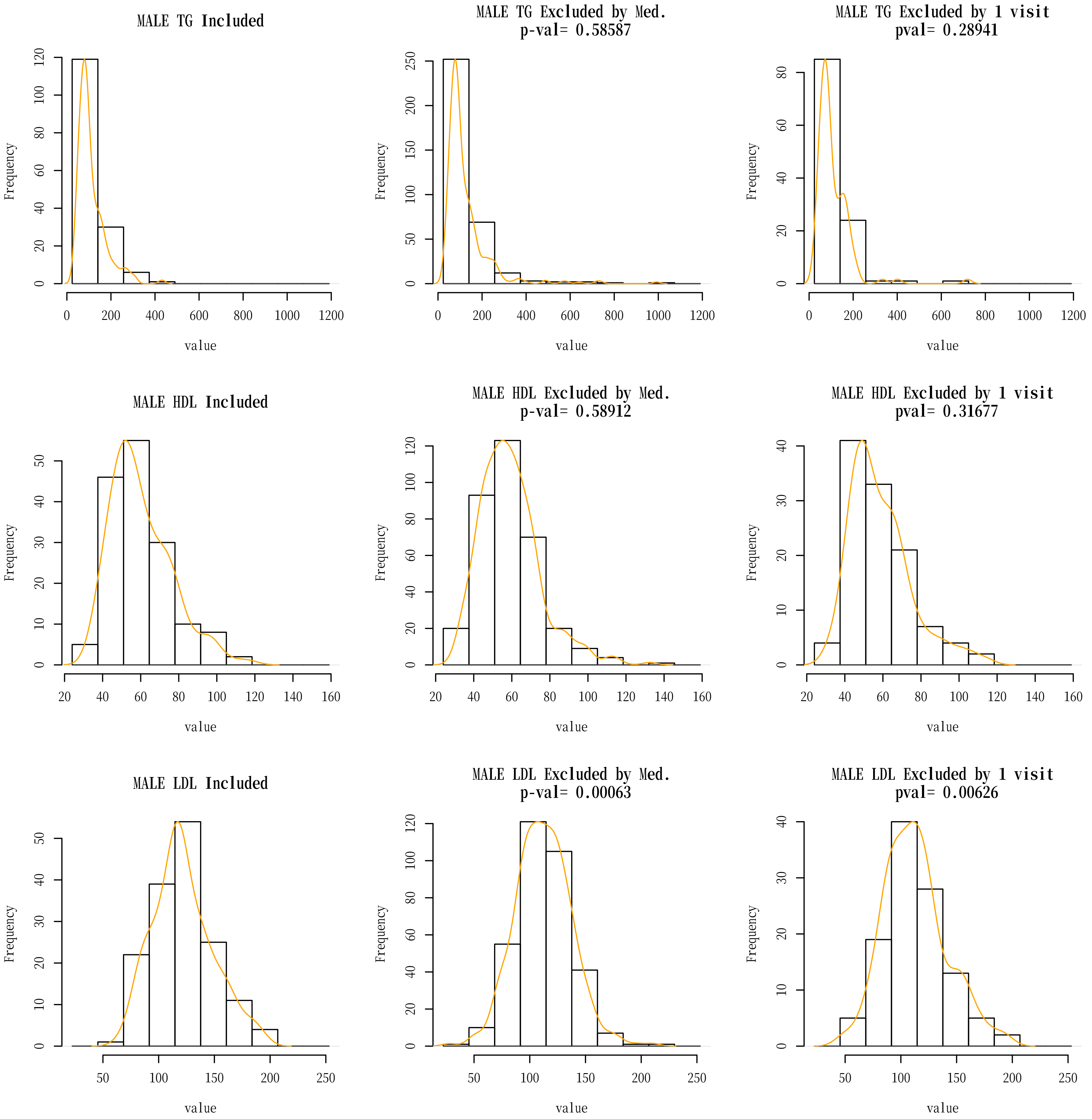
MALE TG Included
Frequency
0 20 40 60 80 100 120
0 200 400 600 800 1000 1200
value
MALE TG Excluded by Med.
p-val= 0.58587
Frequency
0 50 100 150 200 250
0 200 400 600 800 1000 1200
value
MALE TG Excluded by 1 visit
pval= 0.28941
Frequency
0 20 40 60 80
0 200 400 600 800 1000 1200
value
MALE HDL Included
Frequency
0 10 20 30 40 50
20 40 60 80 100 120 140 160
value
MALE HDL Excluded by Med.
p-val= 0.58912
Frequency
0 20 40 60 80 100 120
20 40 60 80 100 120 140 160
value
MALE HDL Excluded by 1 visit
pval= 0.31677
Frequency
0 10 20 30 40
20 40 60 80 100 120 140 160
value
MALE LDL Included
Frequency
0 10 20 30 40 50
50 100 150 200 250
value
MALE LDL Excluded by Med.
p-val= 0.00063
Frequency
0 20 40 60 80 100 120
50 100 150 200 250
value
MALE LDL Excluded by 1 visit
pval= 0.00626
Frequency
0 10 20 30 40
50 100 150 200 250
value

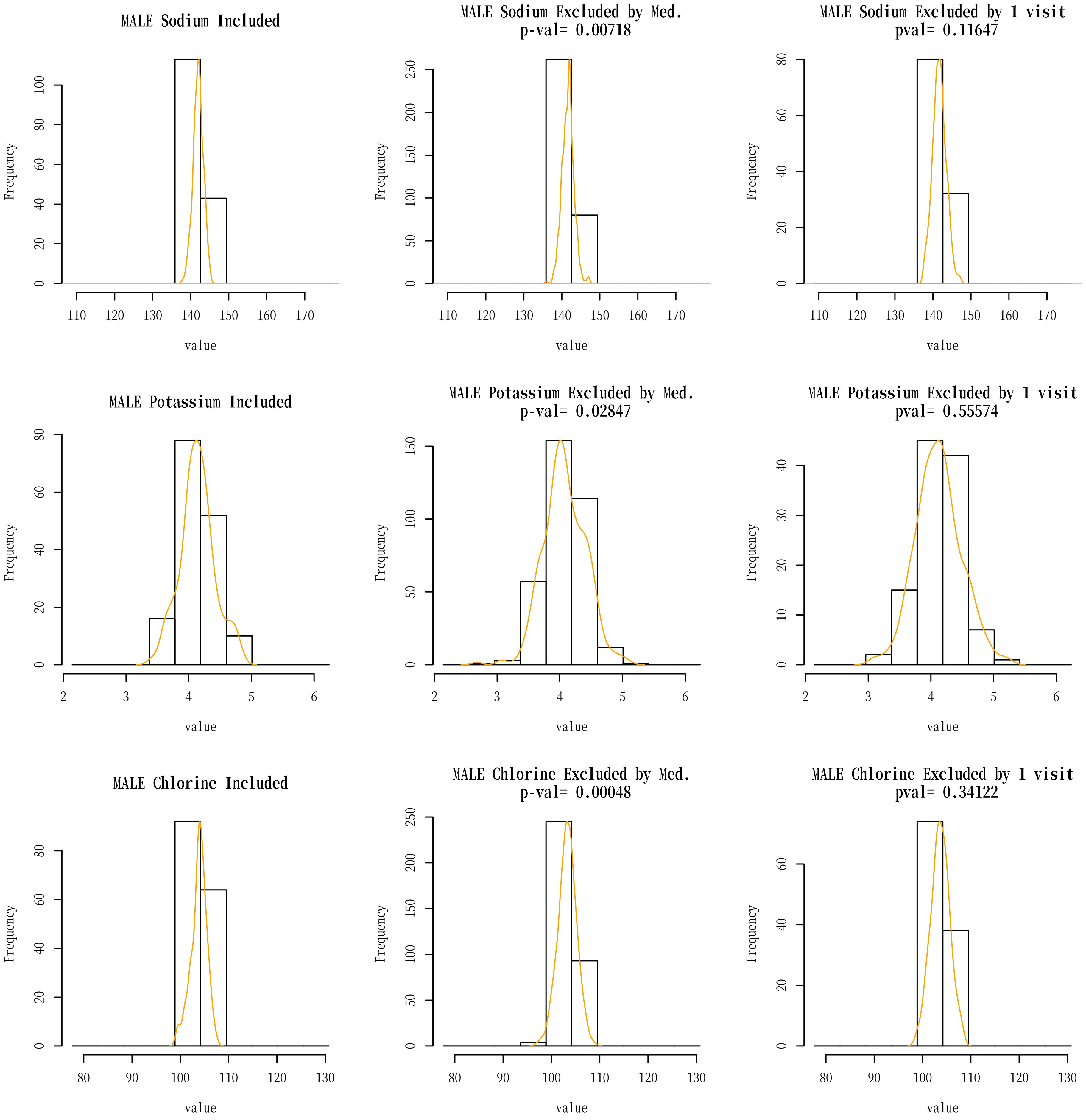
MALE Sodium Included
MALE Sodium Excluded by Med.
p-val= 0.00718
MALE Sodium Excluded by 1 visit
pval= 0.11647
MALE Potassium Included
MALE Potassium Excluded by Med.
p-val= 0.02847
MALE Potassium Excluded by 1 visit
pval= 0.55574
MALE Chlorine Included
MALE Chlorine Excluded by Med.
p-val= 0.00048
MALE Chlorine Excluded by 1 visit
pval= 0.34122
Frequency
value
110 120 130 140 150 160 170
2 3 4 5 6
80 90 100 110 120 130

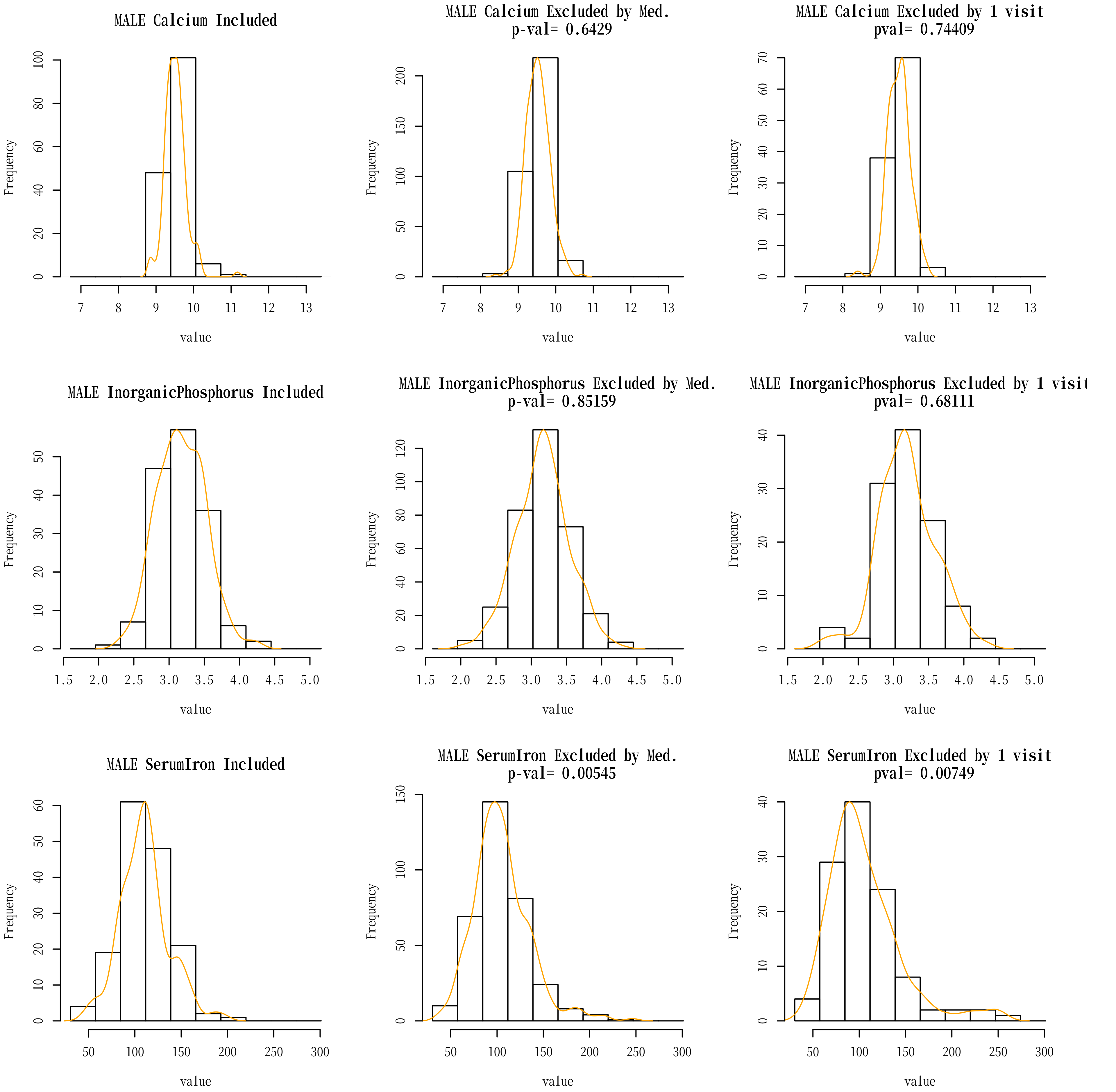
MALE Calcium Included
MALE Calcium Excluded by Med.
p-val= 0.6429
MALE Calcium Excluded by 1 visit
pval= 0.74409
MALE InorganicPhosphorus Included
MALE InorganicPhosphorus Excluded by Med.
p-val= 0.85159
MALE InorganicPhosphorus Excluded by 1 visit
pval= 0.68111
MALE SerumIron Included
MALE SerumIron Excluded by Med.
p-val= 0.00545
MALE SerumIron Excluded by 1 visit
pval= 0.00749
Frequency
value

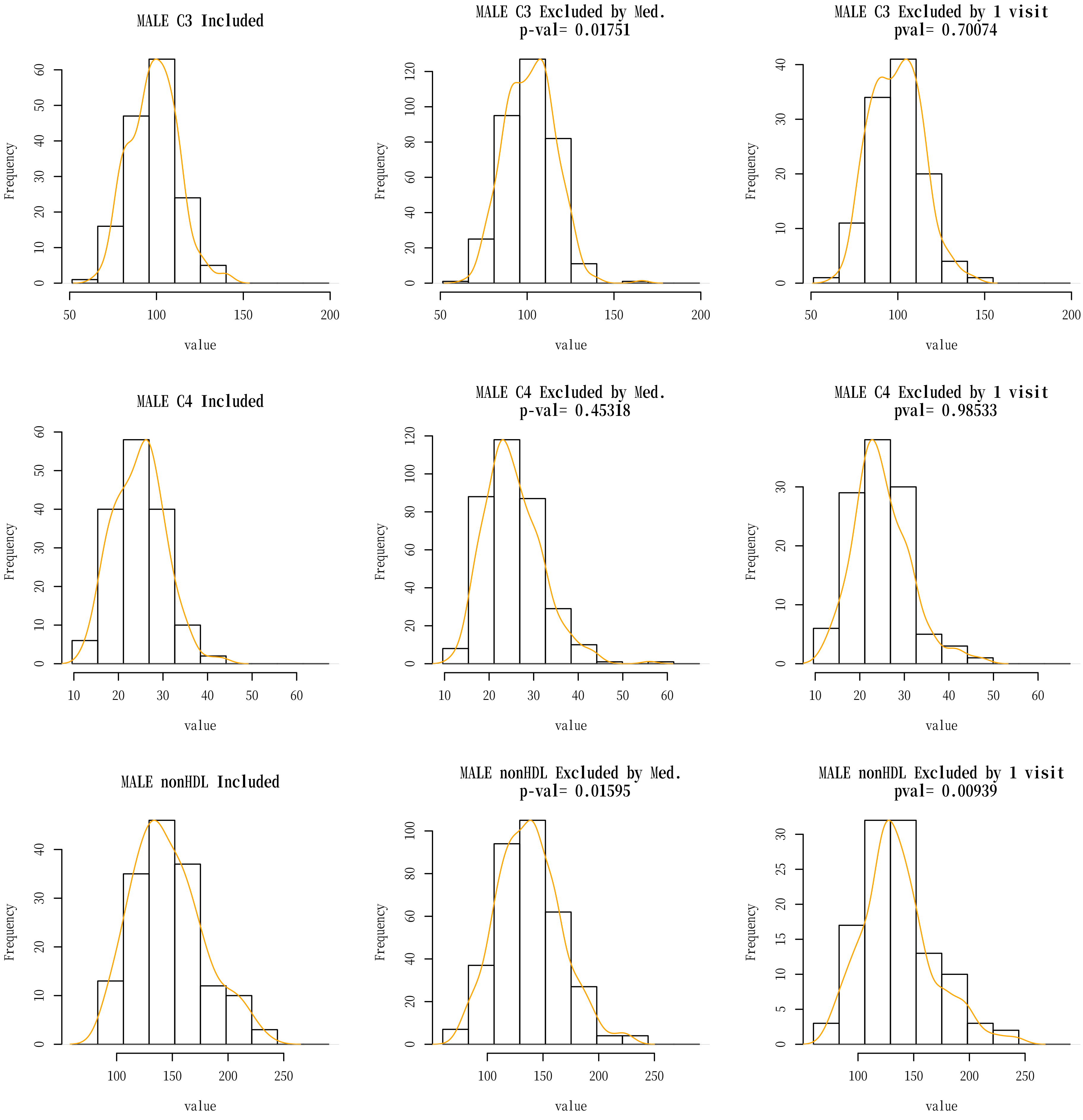

MALE C3 Included
Frequency
value
MALE C3 Excluded by Med.
p-val= 0.01751
Frequency
value
MALE C3 Excluded by 1 visit
pval= 0.70074
Frequency
value
MALE C4 Included
Frequency
value
MALE C4 Excluded by Med.
p-val= 0.45318
Frequency
value
MALE C4 Excluded by 1 visit
pval= 0.98533
Frequency
value
MALE nonHDL Included
Frequency
value
MALE nonHDL Excluded by Med.
p-val= 0.01595
Frequency
value
MALE nonHDL Excluded by 1 visit
pval= 0.00939
Frequency
value

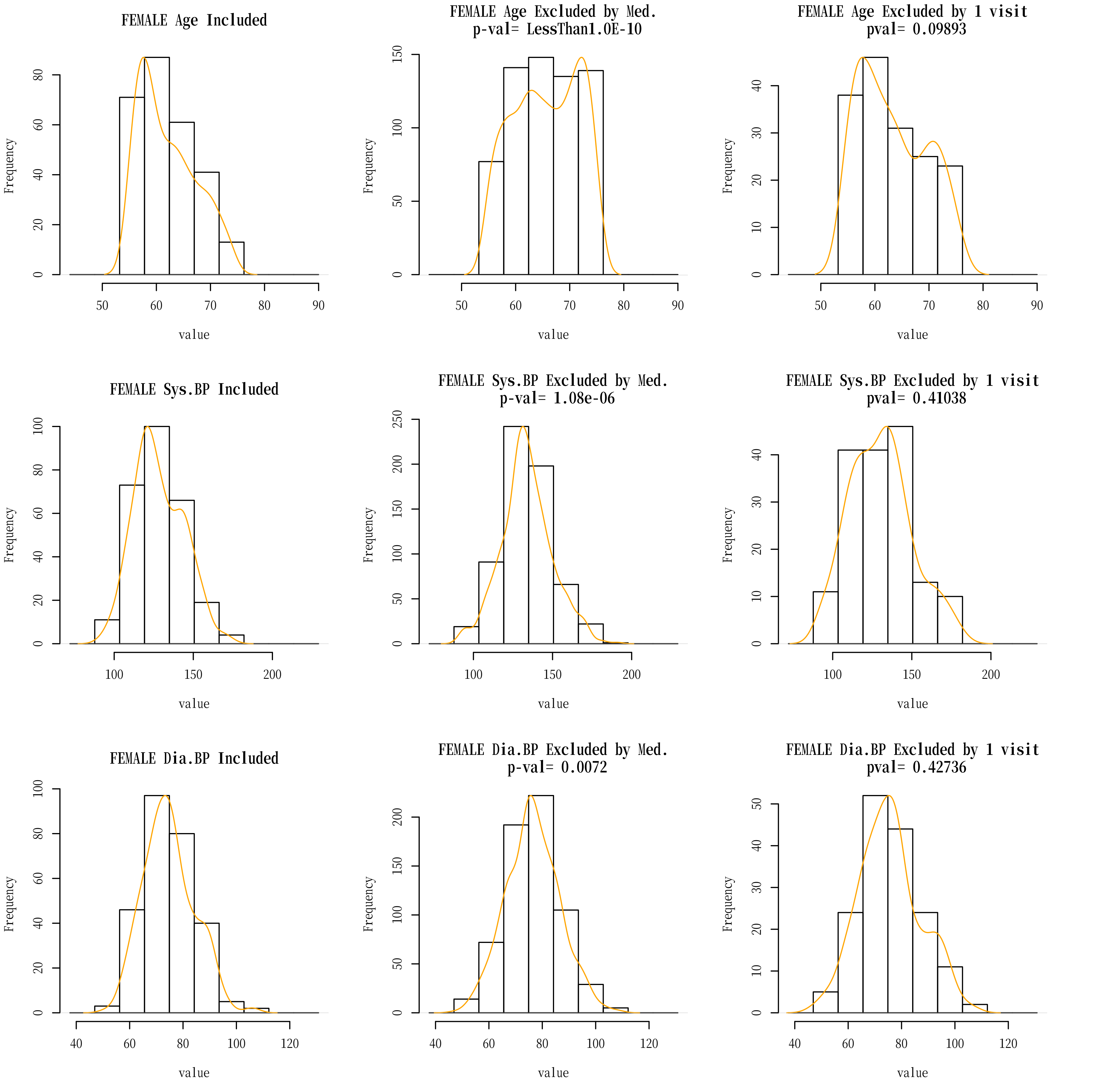
FEMALE Age Included
FEMALE Age Excluded by Med.
p-val= LessThan1.0E-10
FEMALE Age Excluded by 1 visit
pval= 0.09893
FEMALE Sys.BP Included
FEMALE Sys.BP Excluded by Med.
p-val= 1.08e-06
FEMALE Sys.BP Excluded by 1 visit
pval= 0.41038
FEMALE Dia.BP Included
FEMALE Dia.BP Excluded by Med.
p-val= 0.0072
FEMALE Dia.BP Excluded by 1 visit
pval= 0.42736
Frequency
value

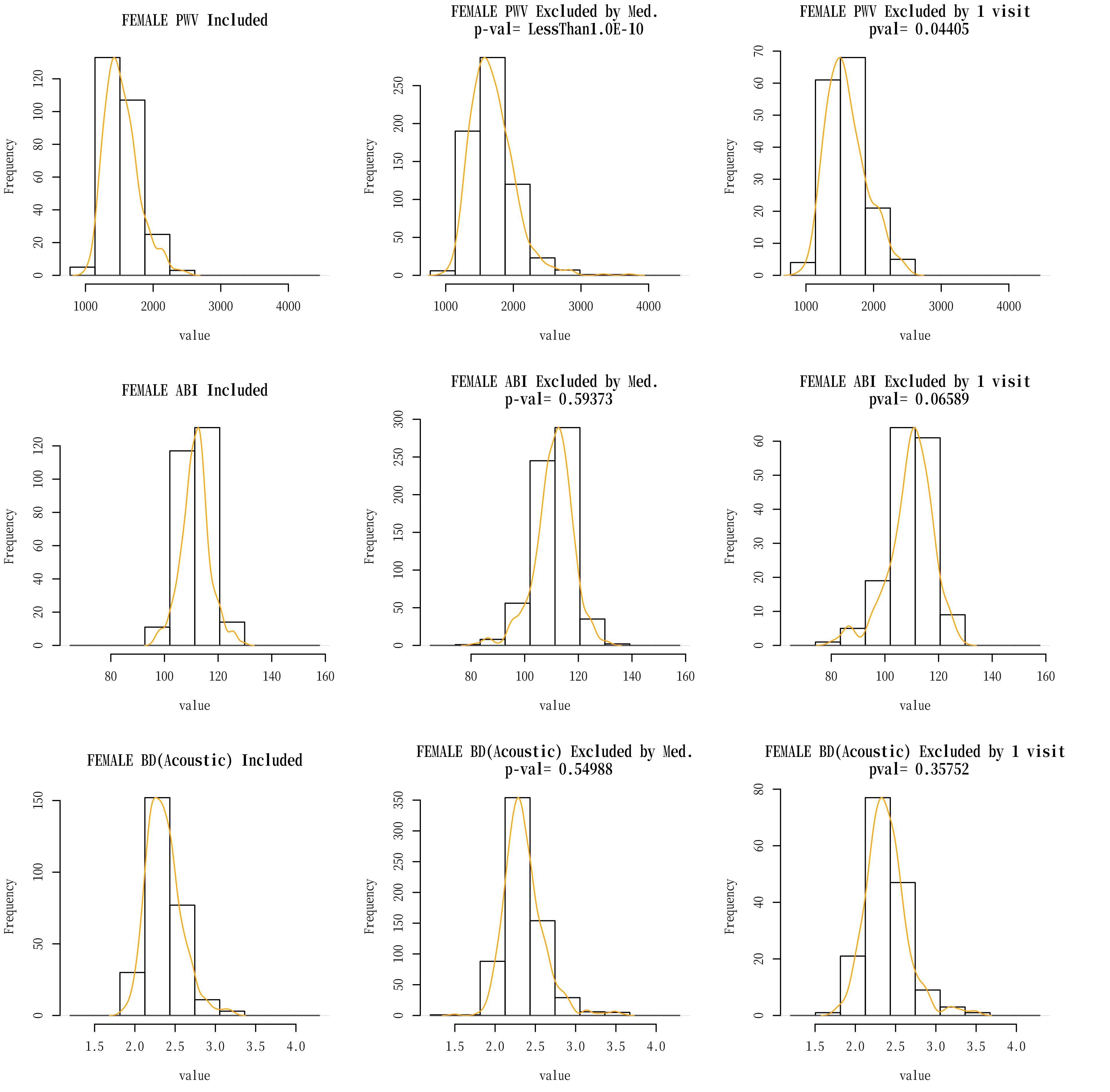
FEMALE PWV Included
Frequency
value
FEMALE PWV Excluded by Med.
p-val= LessThan1.0E-10
Frequency
value
FEMALE PWV Excluded by 1 visit
pval= 0.04405
Frequency
value
FEMALE ABI Included
Frequency
value
FEMALE ABI Excluded by Med.
p-val= 0.59373
Frequency
value
FEMALE ABI Excluded by 1 visit
pval= 0.06589
Frequency
value
FEMALE BD(Acoustic) Included
Frequency
value
FEMALE BD(Acoustic) Excluded by Med.
p-val= 0.54988
Frequency
value
FEMALE BD(Acoustic) Excluded by 1 visit
pval= 0.35752
Frequency
value

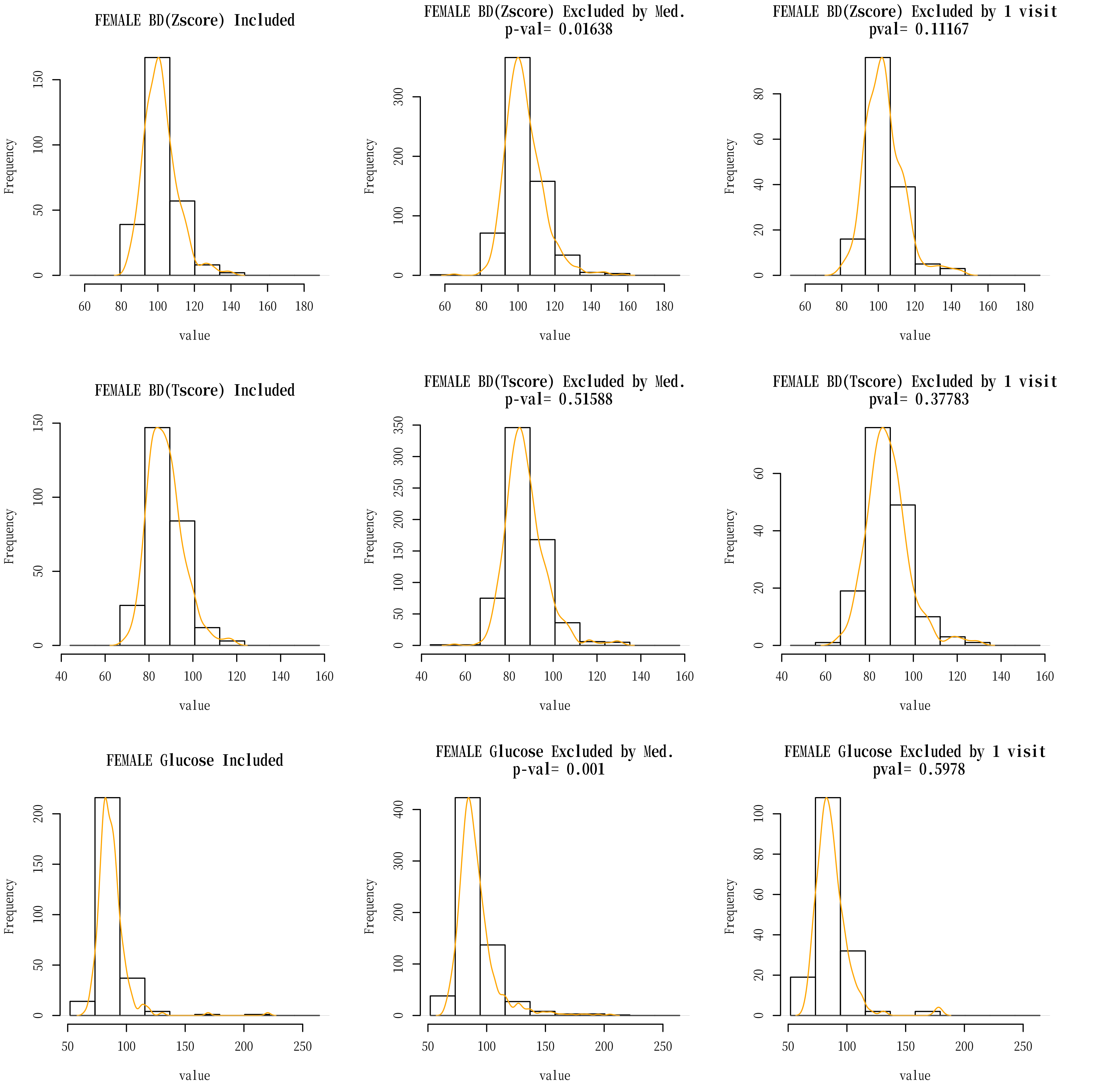
FEMALE BD(Zscore) Included
FEMALE BD(Zscore) Excluded by Med.
p-val= 0.01638
FEMALE BD(Zscore) Excluded by 1 visit
pval= 0.11167
FEMALE BD(Tscore) Included
FEMALE BD(Tscore) Excluded by Med.
p-val= 0.51588
FEMALE BD(Tscore) Excluded by 1 visit
pval= 0.37783
FEMALE Glucose Included
FEMALE Glucose Excluded by Med.
p-val= 0.001
FEMALE Glucose Excluded by 1 visit
pval= 0.5978
Frequency
value
0
50
100
150
200
250
300
350
400
20
40
60
80
120
140
160
180

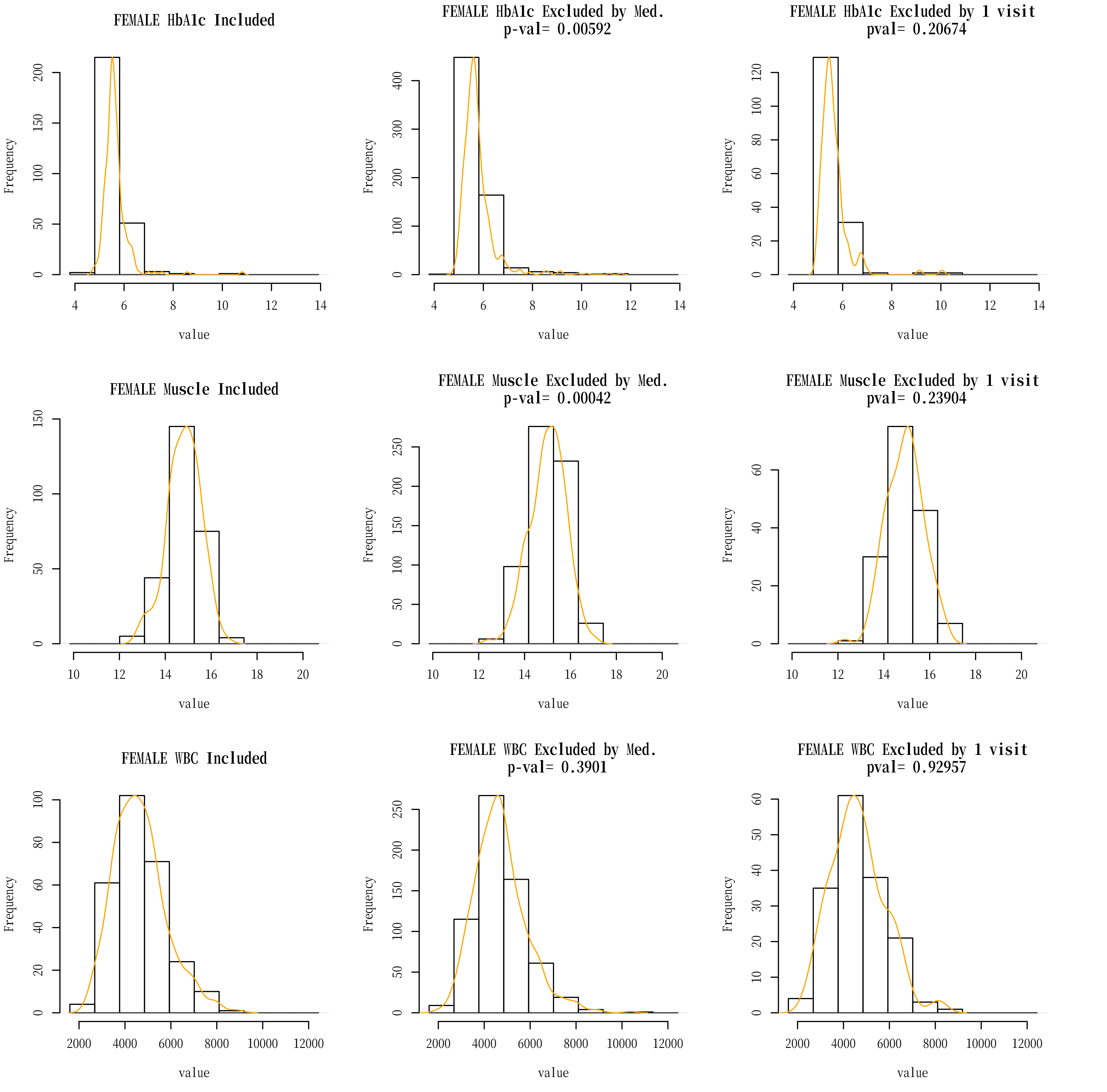
FEMALE HbA1c Included
FEMALE HbA1c Excluded by Med.
p-val= 0.00592
FEMALE HbA1c Excluded by 1 visit
pval= 0.20674
FEMALE Muscle Included
FEMALE Muscle Excluded by Med.
p-val= 0.00042
FEMALE Muscle Excluded by 1 visit
pval= 0.23904
FEMALE WBC Included
FEMALE WBC Excluded by Med.
p-val= 0.3901
FEMALE WBC Excluded by 1 visit
pval= 0.92957
Frequency
value

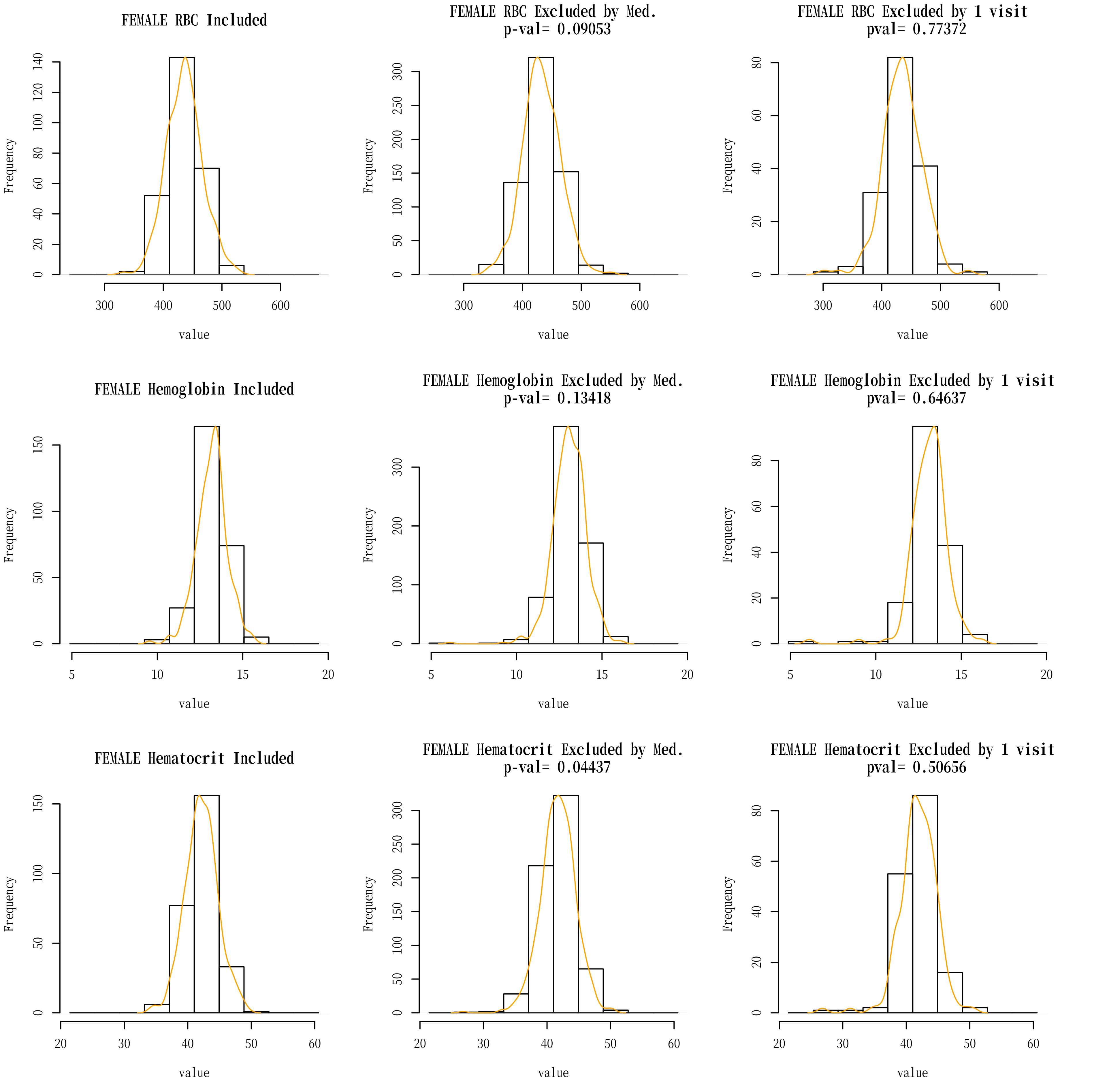
FEMALE RBC Included
FEMALE RBC Excluded by Med.
p-val= 0.09053
FEMALE RBC Excluded by 1 visit
pval= 0.77372
FEMALE Hemoglobin Included
FEMALE Hemoglobin Excluded by Med.
p-val= 0.13418
FEMALE Hemoglobin Excluded by 1 visit
pval= 0.64637
FEMALE Hematocrit Included
FEMALE Hematocrit Excluded by Med.
p-val= 0.04437
FEMALE Hematocrit Excluded by 1 visit
pval= 0.50656
Frequency
value

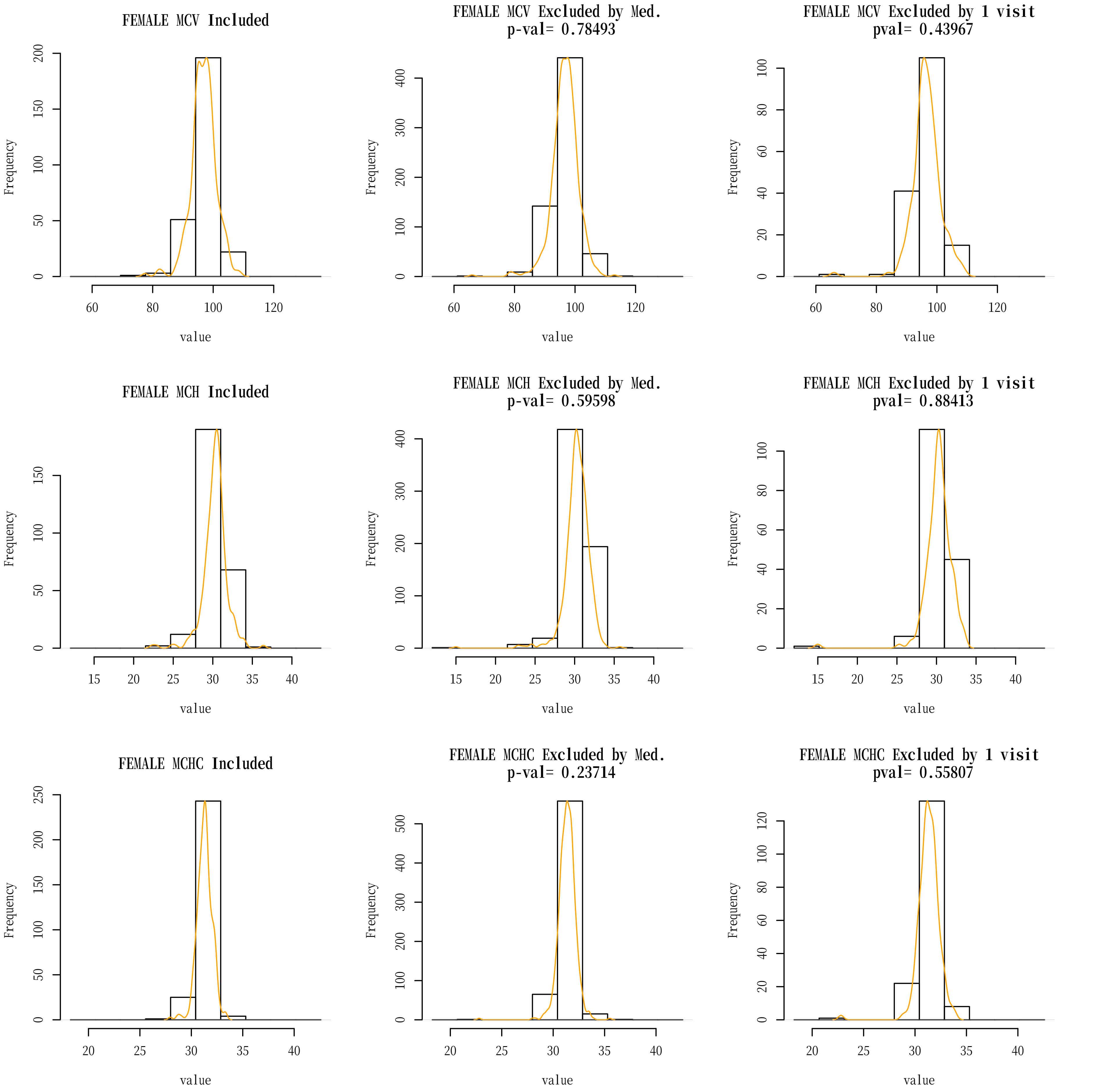
FEMALE MCV Included
FEMALE MCV Excluded by Med.
p-val= 0.78493
FEMALE MCV Excluded by 1 visit
pval= 0.43967
FEMALE MCH Included
FEMALE MCH Excluded by Med.
p-val= 0.59598
FEMALE MCH Excluded by 1 visit
pval= 0.88413
FEMALE MCHC Included
FEMALE MCHC Excluded by Med.
p-val= 0.23714
FEMALE MCHC Excluded by 1 visit
pval= 0.55807
Frequency
value

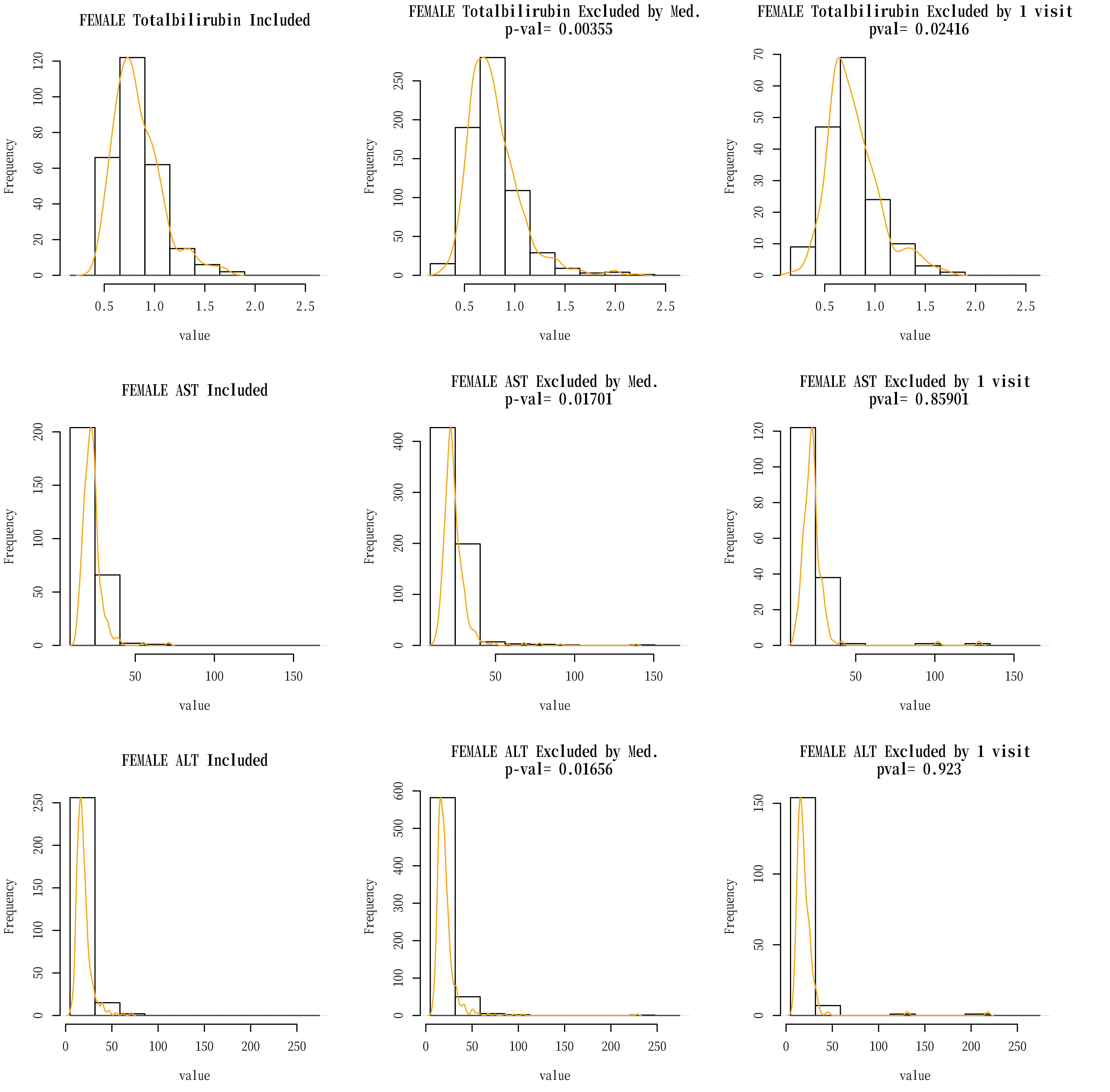
FEMALE Totalbilirubin Included
FEMALE Totalbilirubin Excluded by Med.
p-val= 0.00355
FEMALE Totalbilirubin Excluded by 1 visit
pval= 0.02416
FEMALE AST Included
FEMALE AST Excluded by Med.
p-val= 0.01701
FEMALE AST Excluded by 1 visit
pval= 0.85901
FEMALE ALT Included
FEMALE ALT Excluded by Med.
p-val= 0.01656
FEMALE ALT Excluded by 1 visit
pval= 0.923
Frequency
value

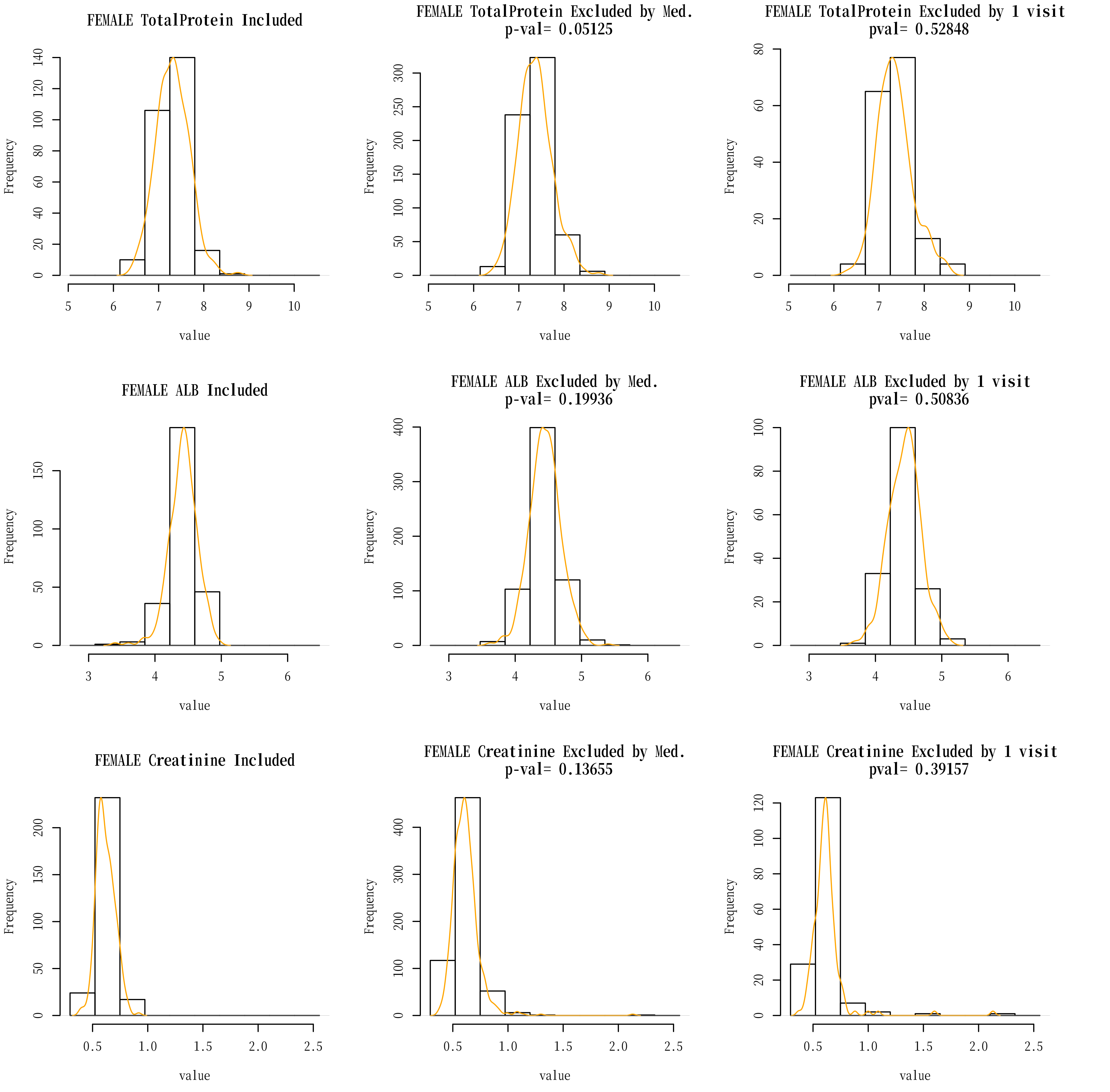
FEMALE TotalProtein Included
FEMALE TotalProtein Excluded by Med.
p-val= 0.05125
FEMALE TotalProtein Excluded by 1 visit
pval= 0.52848
FEMALE ALB Included
FEMALE ALB Excluded by Med.
p-val= 0.19936
FEMALE ALB Excluded by 1 visit
pval= 0.50836
FEMALE Creatinine Included
FEMALE Creatinine Excluded by Med.
p-val= 0.13655
FEMALE Creatinine Excluded by 1 visit
pval= 0.39157
Frequency
value

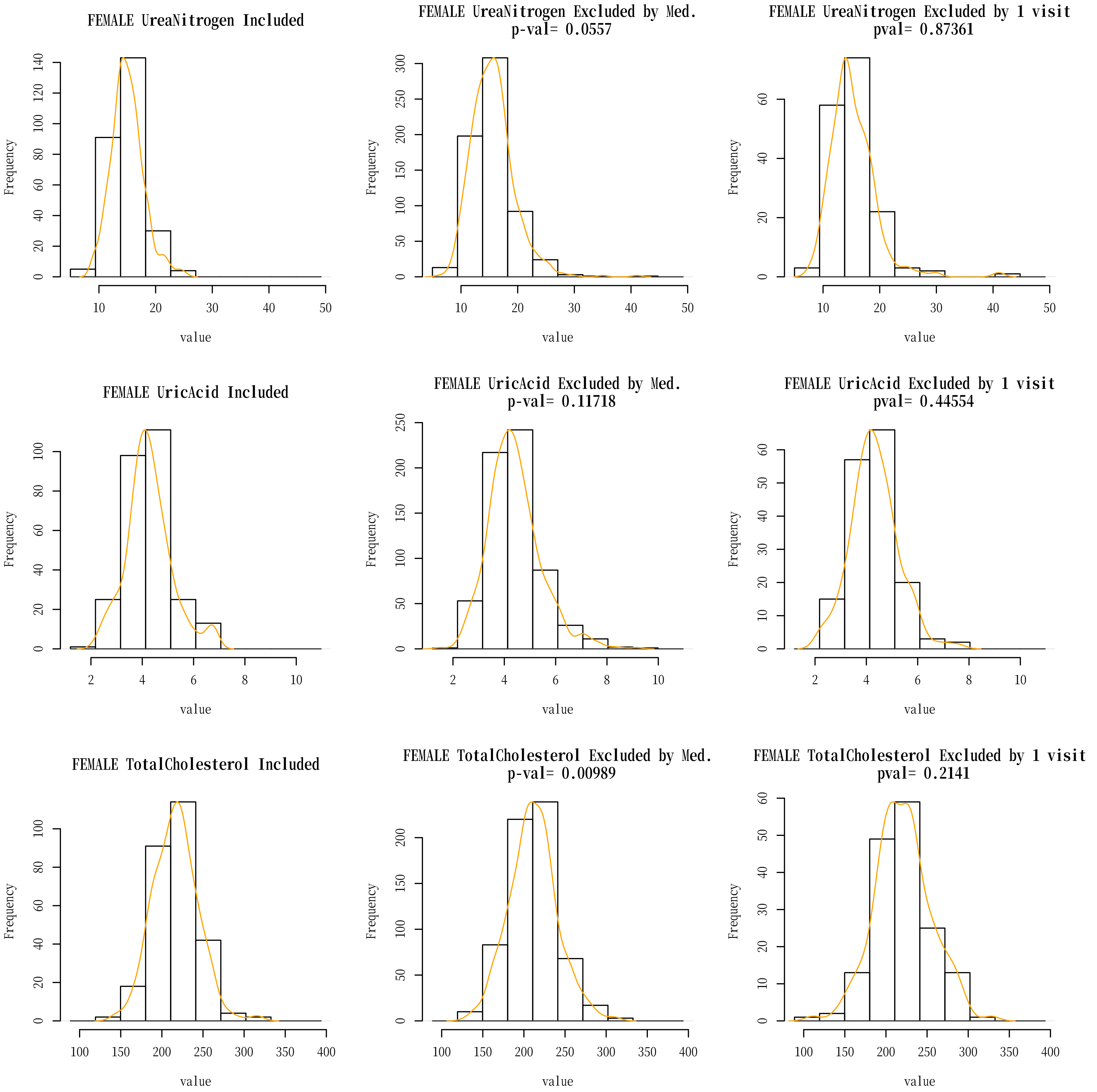

FEMALE UreaNitrogen Included
FEMALE UreaNitrogen Excluded by Med.
p-val= 0.0557
FEMALE UreaNitrogen Excluded by 1 visit
pval= 0.87361
FEMALE UricAcid Included
FEMALE UricAcid Excluded by Med.
p-val= 0.11718
FEMALE UricAcid Excluded by 1 visit
pval= 0.44554
FEMALE TotalCholesterol Included
FEMALE TotalCholesterol Excluded by Med.
p-val= 0.00989
FEMALE TotalCholesterol Excluded by 1 visit
pval= 0.2141
Frequency
value

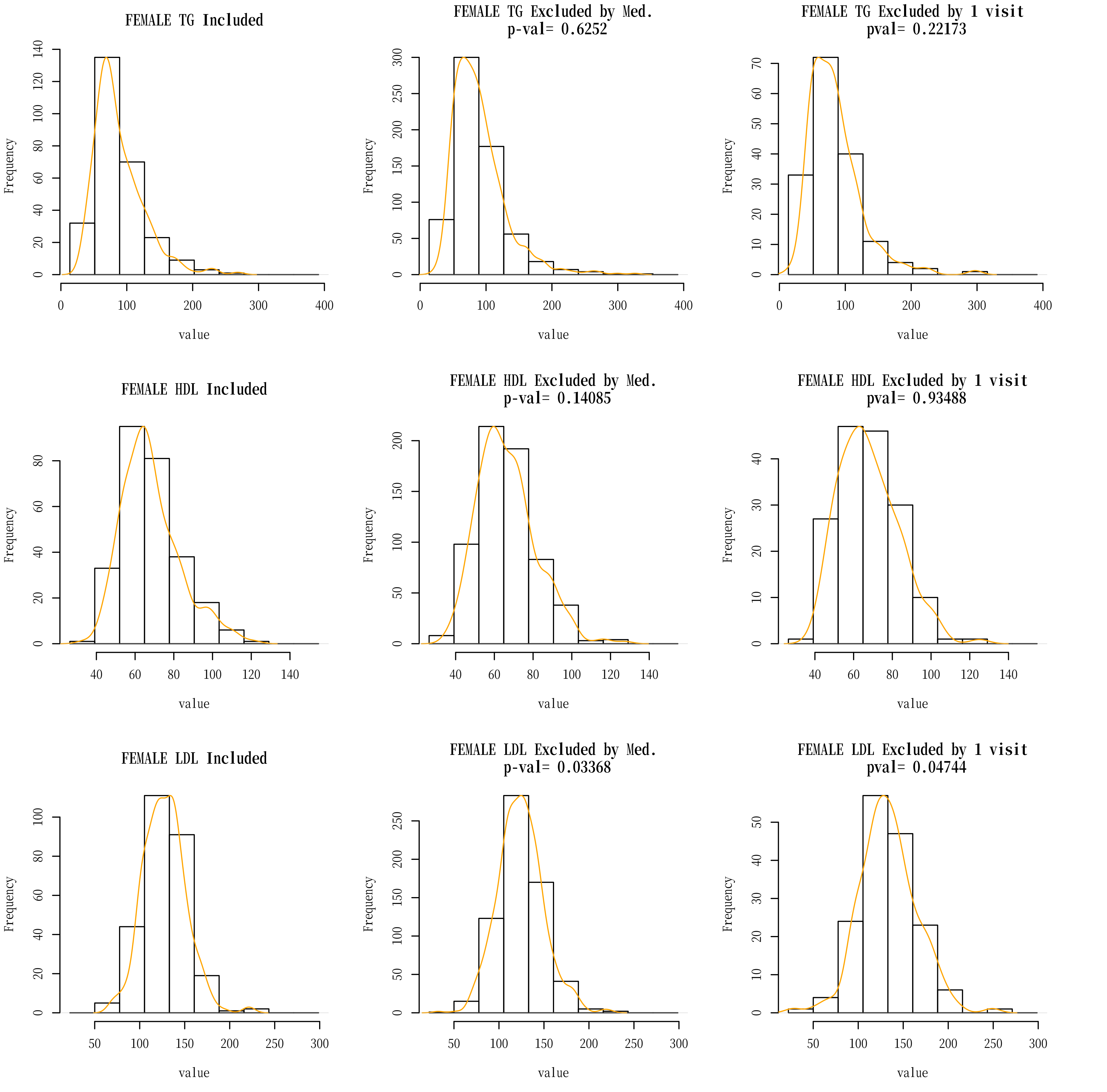
FEMALE TG Included
FEMALE TG Excluded by Med.
p-val= 0.6252
FEMALE TG Excluded by 1 visit
pval= 0.22173
FEMALE HDL Included
FEMALE HDL Excluded by Med.
p-val= 0.14085
FEMALE HDL Excluded by 1 visit
pval= 0.93488
FEMALE LDL Included
FEMALE LDL Excluded by Med.
p-val= 0.03368
FEMALE LDL Excluded by 1 visit
pval= 0.04744
Frequency
value
0 100 200 300 400
40 60 80 100 120 140
50 100 150 200 250 300
0 20 40 60 80 100 120 140
0 50 100 150 200 250 300
0 10 20 30 40 50 60 70

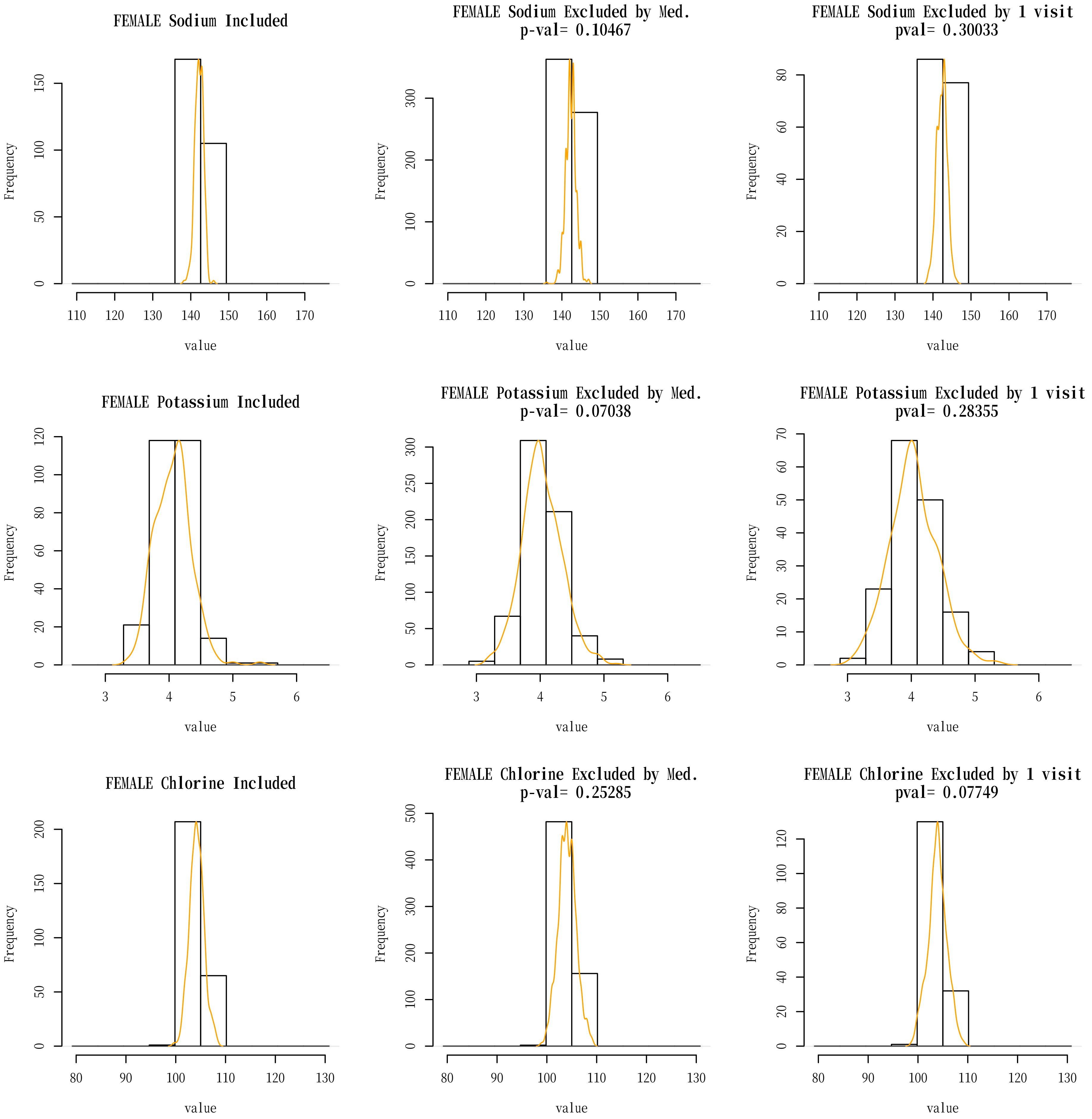
FEMALE Sodium Included
FEMALE Sodium Excluded by Med.
p-val= 0.10467
FEMALE Sodium Excluded by 1 visit
pval= 0.30033
FEMALE Potassium Included
FEMALE Potassium Excluded by Med.
p-val= 0.07038
FEMALE Potassium Excluded by 1 visit
pval= 0.28355
FEMALE Chlorine Included
FEMALE Chlorine Excluded by Med.
p-val= 0.25285
FEMALE Chlorine Excluded by 1 visit
pval= 0.07749
Frequency
value

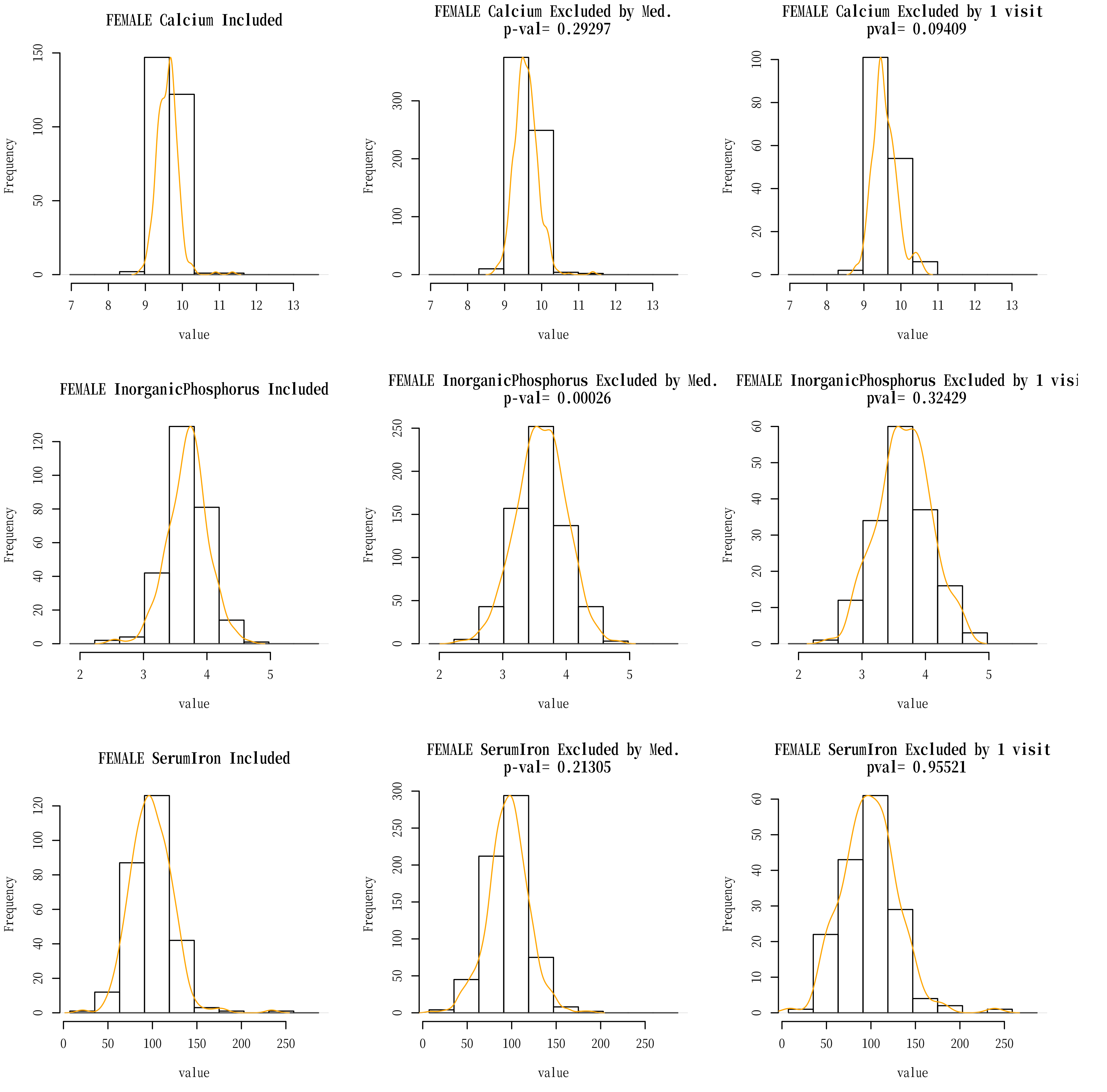
FEMALE Calcium Included
Frequency
value
FEMALE Calcium Excluded by Med.
p-val= 0.29297
Frequency
value
FEMALE Calcium Excluded by 1 visit
pval= 0.09409
Frequency
value
FEMALE InorganicPhosphorus Included
Frequency
value
FEMALE InorganicPhosphorus Excluded by Med.
p-val= 0.00026
Frequency
value
FEMALE InorganicPhosphorus Excluded by 1 visi
pval= 0.32429
Frequency
value
FEMALE SerumIron Included
Frequency
value
FEMALE SerumIron Excluded by Med.
p-val= 0.21305
Frequency
value
FEMALE SerumIron Excluded by 1 visit
pval= 0.95521
Frequency
value

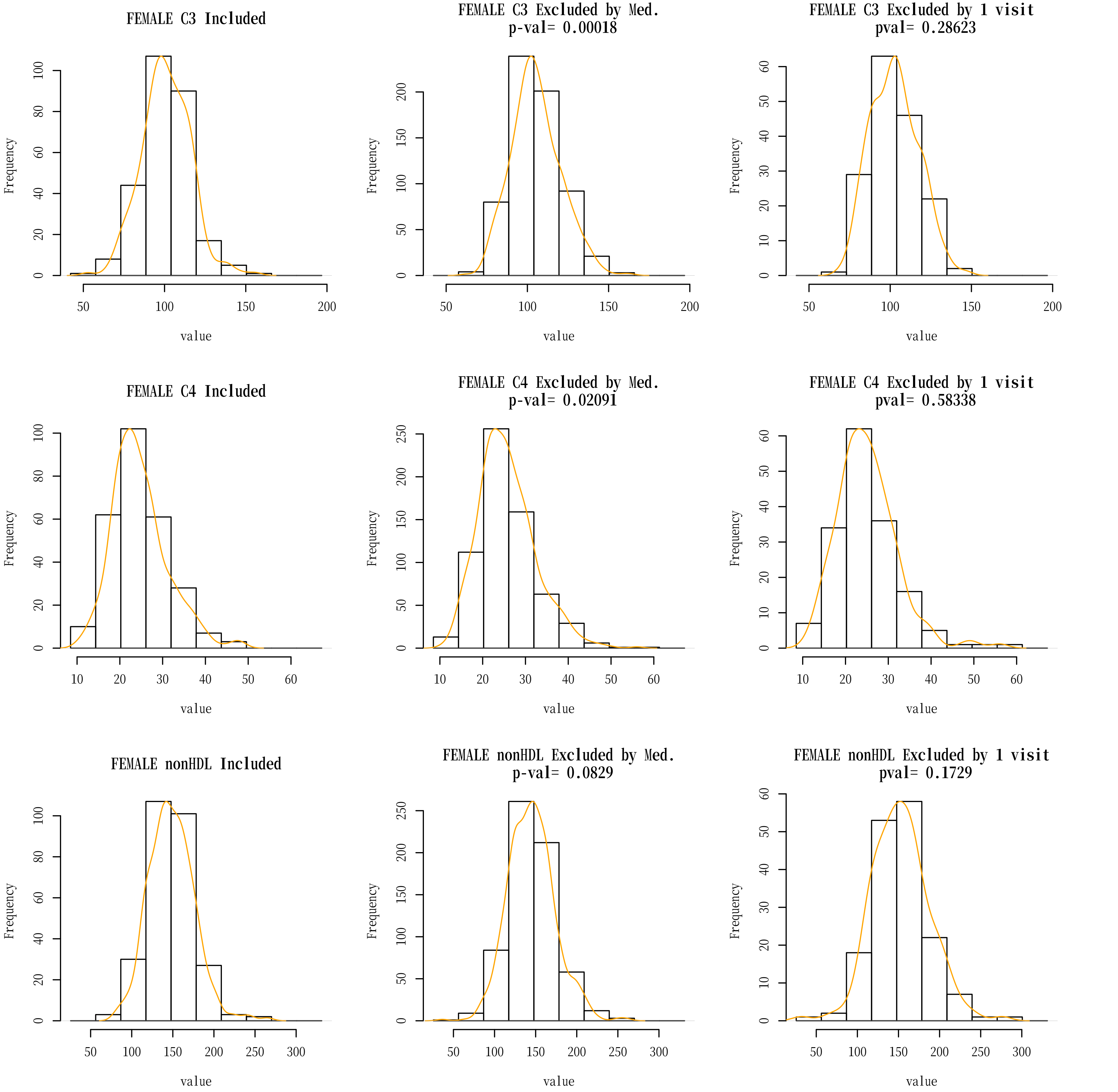
FEMALE C3 Included
FEMALE C3 Excluded by Med.
p-val= 0.00018
FEMALE C3 Excluded by 1 visit
pval= 0.28623
FEMALE C4 Included
FEMALE C4 Excluded by Med.
p-val= 0.02091
FEMALE C4 Excluded by 1 visit
pval= 0.58338
FEMALE nonHDL Included
FEMALE nonHDL Excluded by Med.
p-val= 0.0829
FEMALE nonHDL Excluded by 1 visit
pval= 0.1729
Frequency
value
