# Supplementary figures and images for: Prediction of blood test values under different lifestyle scenarios using time-series electronic health record
Source: PLoS One. 2020 Mar 20;15(3):e0230172. doi: 10.1371/journal.pone.0230172 (PMC7083324; doi:10.1371/journal.pone.0230172)

# MALE 55-75

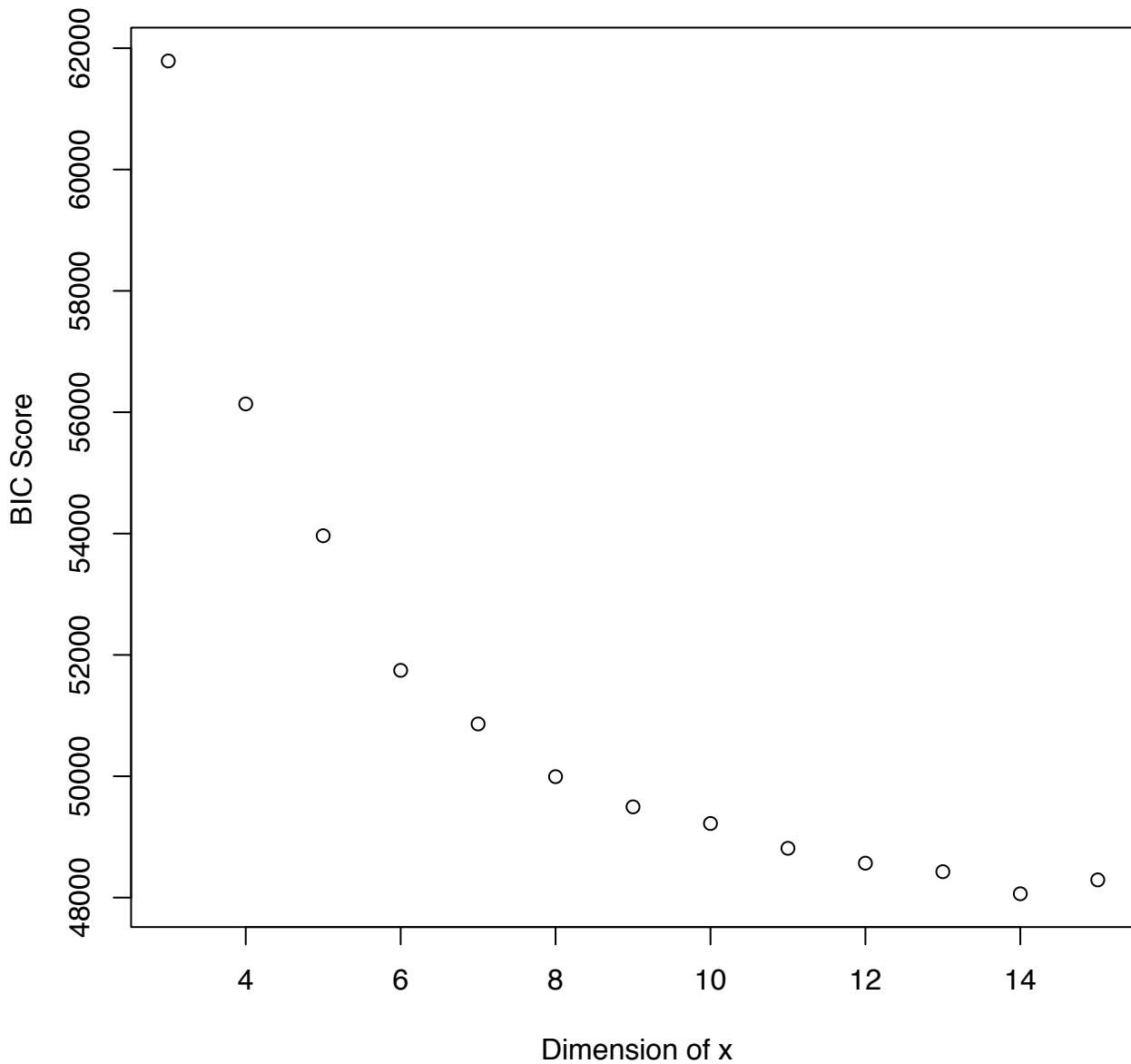

## FEMALE 55-75

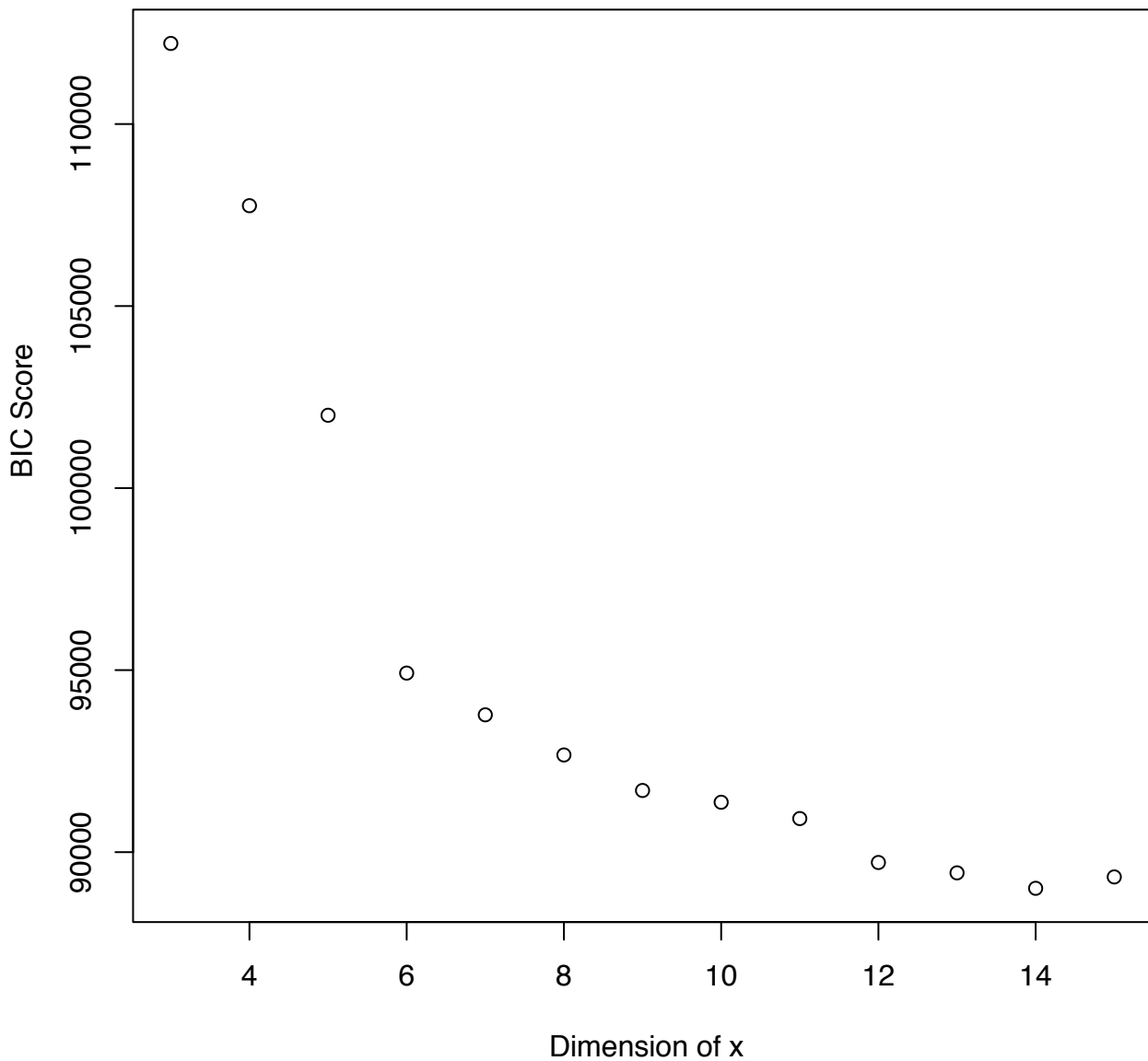

Supplement: S2 Fig — The lowest BIC score of each system dimension p (p = 1, …, 15) for both dataset. (PDF) [file pone.0230172.s003.pdf]

# MALE from 55 to 75

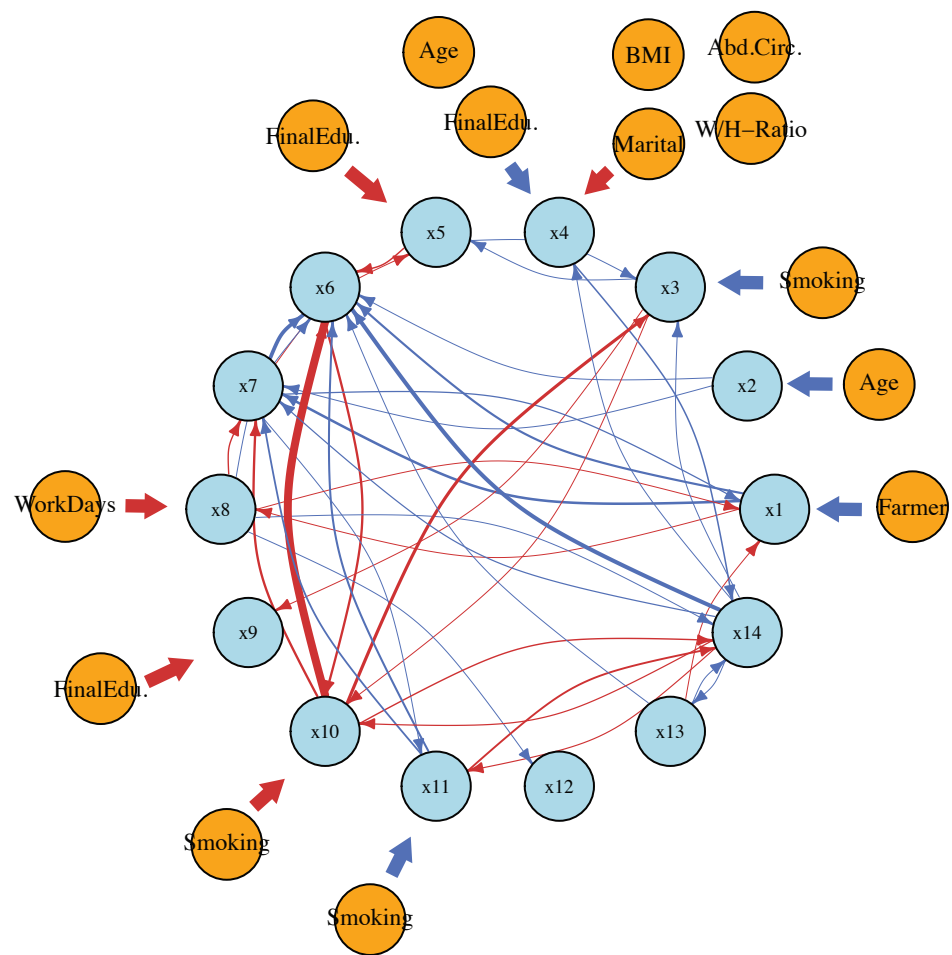

# FEMALE from 55 to 75

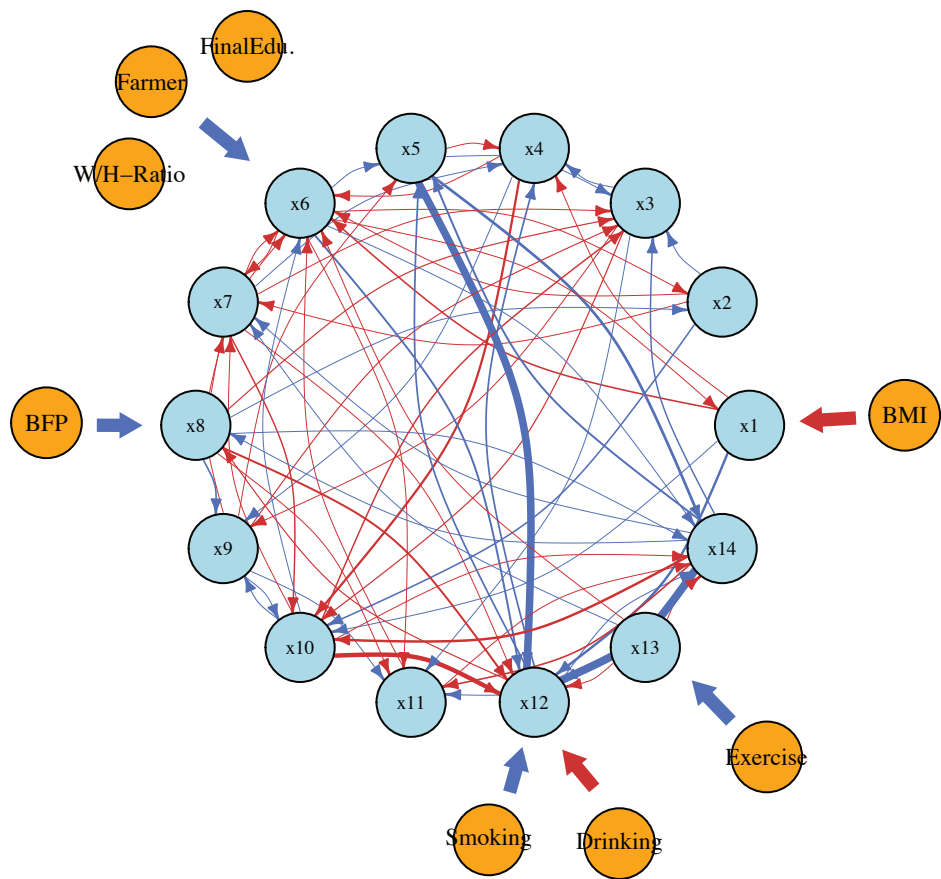

Supplement: S3 Fig — The regulatory relationships among xk,t and zk,t generated from F for both dataset. (PDF) [file pone.0230172.s004.pdf]
